# Evolutionary trade-offs constraining the MHC gene expansion: beyond simple TCR depletion model

**DOI:** 10.3389/fimmu.2023.1240723

**Published:** 2024-01-08

**Authors:** Magdalena Migalska, Kazimierz Węglarczyk, Katarzyna Dudek, Joanna Homa

**Affiliations:** ^1^ Institute of Environmental Sciences, Faculty of Biology, Jagiellonian University, Krakow, Poland; ^2^ Department of Clinical Immunology, Institute of Paediatrics, Jagiellonian University Medical College, Krakow, Poland; ^3^ Department of Evolutionary Immunology, Institute of Zoology and Biomedical Research, Faculty of Biology, Jagiellonian University, Krakow, Poland

**Keywords:** major histocompatibility complex, T-cell receptor, evolutionary trade-off, *Myodes glareolus*, adaptive immunity

## Abstract

The immune system is as much shaped by the pressure of pathogens as it is by evolutionary trade-offs that constrain its structure and function. A perfect example comes from the major histocompatibility complex (MHC), molecules that initiate adaptive immune response by presentation of foreign antigens to T cells. The remarkable, population-level polymorphism of MHC genes is assumed to result mainly from a co-evolutionary arms race between hosts and pathogens, while the limited, within-individual number of functional MHC loci is thought to be the consequence of an evolutionary trade-off between enhanced pathogen recognition and excessive T cell depletion during negative selection in the thymus. Certain mathematical models and infection studies suggest that an intermediate individual MHC diversity would thus be optimal. A recent, more direct test of this hypothesis has shown that the effects of MHC diversity on T-cell receptor (TCR) repertoires may differ between MHC classes, supporting the depletion model only for MHC class I. Here, we used the bank vole (*Myodes=Cletronomys glareolus*), a rodent species with variable numbers of expressed MHC genes, to test how an individual MHC diversity influences the proportions and TCR repertoires of responding T cell subsets. We found a non-linear relationship between MHC diversity and T cell proportions (with intermediate MHC numbers coinciding with the largest T cell proportions), perhaps reflecting an optimality effect of balanced positive and negative thymic selection. The association was strongest for the relationship between MHC class I and splenic CD8+ T cells. The CD8+ TCR richness alone was unaffected by MHC class I diversity, suggesting that MHC class I expansion may be limited by decreasing T cell counts, rather than by direct depletion of TCR richness. In contrast, CD4+ TCR richness was positively correlated with MHC class II diversity, arguing against a universal TCR depletion. It also suggests that different evolutionary forces or trade-offs may limit the within-individual expansion of the MHC class II loci.

## 1 Introduction

The astounding complexity of the vertebrate immune system was largely shaped by the pathogen pressure, but neither structure nor function of this system can be fully understood without accounting for trade-offs that constrained its evolution. The major histocompatibility complex (MHC) perfectly illustrates these aspects. Classical MHC molecules (hereafter referred to as “MHC”) are essential for molecular-level self/non-self-recognition and initiation of the adaptive immune response through binding and presentation of foreign antigens to αβ T cells (1). Early on, a bewildering polymorphism of the genes encoding MHC caught the attention of evolutionary biologists, and inspired decades of research that have now established the coevolutionary arms race between hosts and pathogens as one of the driving forces behind this diversity (2). Nevertheless, several key questions remain open (3). For example, while dozens to thousands of MHC alleles segregate in natural populations [polymorphism maintained by various forms of balancing selection, such as heterozygote advantage (4)], individuals possess but a few, functional loci that can accommodate only a small fraction of the adaptive diversity present at the population level. Why, then, do mechanisms that promote large allelic polymorphisms not support MHC gene duplication, which would lead to greater individual-level MHC diversity? The most popular explanation of this apparent paradox invokes an evolutionary trade-off between an increased capacity to recognize pathogens and a compromised immune response caused by mechanisms that maintain immune self-tolerance (5).

This idea was formalized by Nowak et al. (6) (who coined the concept of an *optimal* number of MHC molecules per individual), and was later redefined as a *T cell repertoire depletion* hypothesis (7). It postulates that benefit of a hypothetical MHC expansion within the genome, that is, an enhanced ability to bind and present pathogenic antigens, would be counterbalanced by a drastic reduction in the T-cell receptor (TCR) repertoire during thymic negative selection. The trade-off arises because the primary response of the immune system to these antigens requires recognition by a TCR. Crucially, the anticipatory diversity of the antigen-recognizing portion of each TCR (i.e., complementarity determining region 3, CDR3) is generated by a somatic recombination that brings together different variants of specific gene segments (V and J in the α chain; V, D and J in the β chain) and adds and/or removes random nucleotides at the segment junctions (8). Large random component of this diversity-generating process results in a majority of TCRs being either non-responsive or self-reactive. To ensure both MHC-restriction (i.e., recognition of antigens presented by the MHC, but not of “free” antigens) and tolerance to self-antigens, newly generated TCRs are censored by positive and negative selection in the thymus (9). Positive selection promotes survival of T cells capable of properly interacting with MHC-self-peptide complexes, whereas lack of such interaction leads to death of neglect. During negative selection, T cells bearing TCRs that bind MHC-self-peptides with a too strong an avidity are deleted, or, alternatively, some differentiate into a population of natural regulatory CD4+Foxp3+ Tregs (9). In essence, the T cell repertoire depletion hypothesis proposes that the broader spectrum of peptides bound by an expanded, individual MHC collection would include self-peptides, thus rendering a greater proportion of T cells self-reactive. In turn, excessive number of T cells would be removed in the thymus during negative selection, resulting in a purged TCR repertoire. Consequently, even though additional MHC molecules could present more pathogen-derived antigens, the immune system would have few T cells and TCR types left to respond.

Despite its ingenious allure, T cell repertoire depletion hypothesis has been criticized, pointing out that the additional MHC variants should first promote cell survival during positive selection (10). Deciding which process would dominate is far from intuitive, as several key parameters and assumptions underlying these mathematical models remain uncertain (11, 12). At the most fundamental level, it is still not fully understood to what extent the positive selection is driven by an interaction between variable, but germline-encoded parts of a TCR (i.e. CDR1, CDR2) and relatively conserved (and highly similar among different alleles) parts of MHC molecules, versus by an interaction between hypervariable CDR3 and a peptide alongside the highly polymorphic peptide-binding cleft of the MHC molecules (13–15). The two competing theories favor either a dominant role of an evolutionary, intrinsic predisposition of the TCR to recognize MHC molecules via germline-encoded motifs (16–18), or an instrumental role of the thymic selection in determining MHC-restricted peptide recognition (19, 20). If the latter scenario is correct, an increased individual MHC diversity could indeed enhance positive selection. Finally, even the estimates of the toll that positive and negative selection take on thymocytes vary among studies [although a general consensus is that about 20-25% of cells are positively selected, of which 20-50% survive negative selection; reviewed in (11)].

The evident uncertainty of model parameters renders the question of MHC optimality an empirical one. Evolutionary biologists and ecologists have attempted to address the issue of a limited, individual MHC gene number by studying species characterized by an intra-specific variation in the number of MHC genes. This phenomenon, often referred to as a copy-number variation (CNV), pertains to a situation when haplotypes with different number of duplicated and diverged MHC loci segregate in populations. Animals with an intermediate (and presumably optimal) number of MHC loci were predicted to have the highest immunocompetence (e.g., lowest parasite load or diversity) or to perform best on various measures of fitness (e.g. reproductive success or body condition). However, these indirect tests have yielded mixed results (21–25).

In a recent study, Migalska et al. (26) have provided a first direct test of the key predictions of the TCR depletion hypothesis, by correlating MHC diversity with the TCR repertoire in the bank vole, *Myodes=Cletronomys glareolus*, a species characterized by an extensive inter-individual CNV in MHC genes (27–29). The reported negative correlation between estimates of a total, splenic TCRβ repertoire and the MHC class I diversity partially supported the TCR depletion model, but lack of a significant correlation for MHC class II challenged its generality (26). Importantly, this finding raised new questions about the nature of the trade-offs shaping the evolution of the MHC, as the idea that the diversity of the two classes might affect TCRs differently had never been considered. While both MHC classes present peptides to T cells, they differ markedly in e.g., antigen source and processing pathway, tissue distribution and T cell types they interact with. MHC class I is expressed on all nucleated cells, and generally presents cytosolic peptides (e.g., self-derived under normal conditions, pathogen-derived during infection with viruses, cancer-derived during cancerous transformation) to cytotoxic, CD8+ T cells (1). In contrast, MHC class II is found on specialized, antigen presenting cells (APCs, e.g. dendritic cells, B cells, macrophages), and generally presents exogenous peptides that entered a cell via an endocytic pathway (e.g., phagocytosis or receptor-mediated endocytosis) (30). APCs present such antigens to helper CD4+ T cells, which aid various other immune cells in their tasks (e.g., B cells in antibody production, cytotoxic T cells in growth and activation, macrophages and neutrophils in phagocytosis) (1). The study by Migalska et al. (26) was aimed as an overall test of the TCR depletion hypothesis, and so the methodology used (extraction of RNA from whole spleens) did not allow to disentangle mechanisms behind the discovered discrepancy. In particular, it prevented the actual partitioning of TCR diversity into cellular subsets interacting with MHC class I and class II, respectively. There was also no information on the proportions of e.g., cytotoxic, helper or regulatory T cells, which represent major, functional T cell subsets emerging from thymic selection.

**Figure 1:**
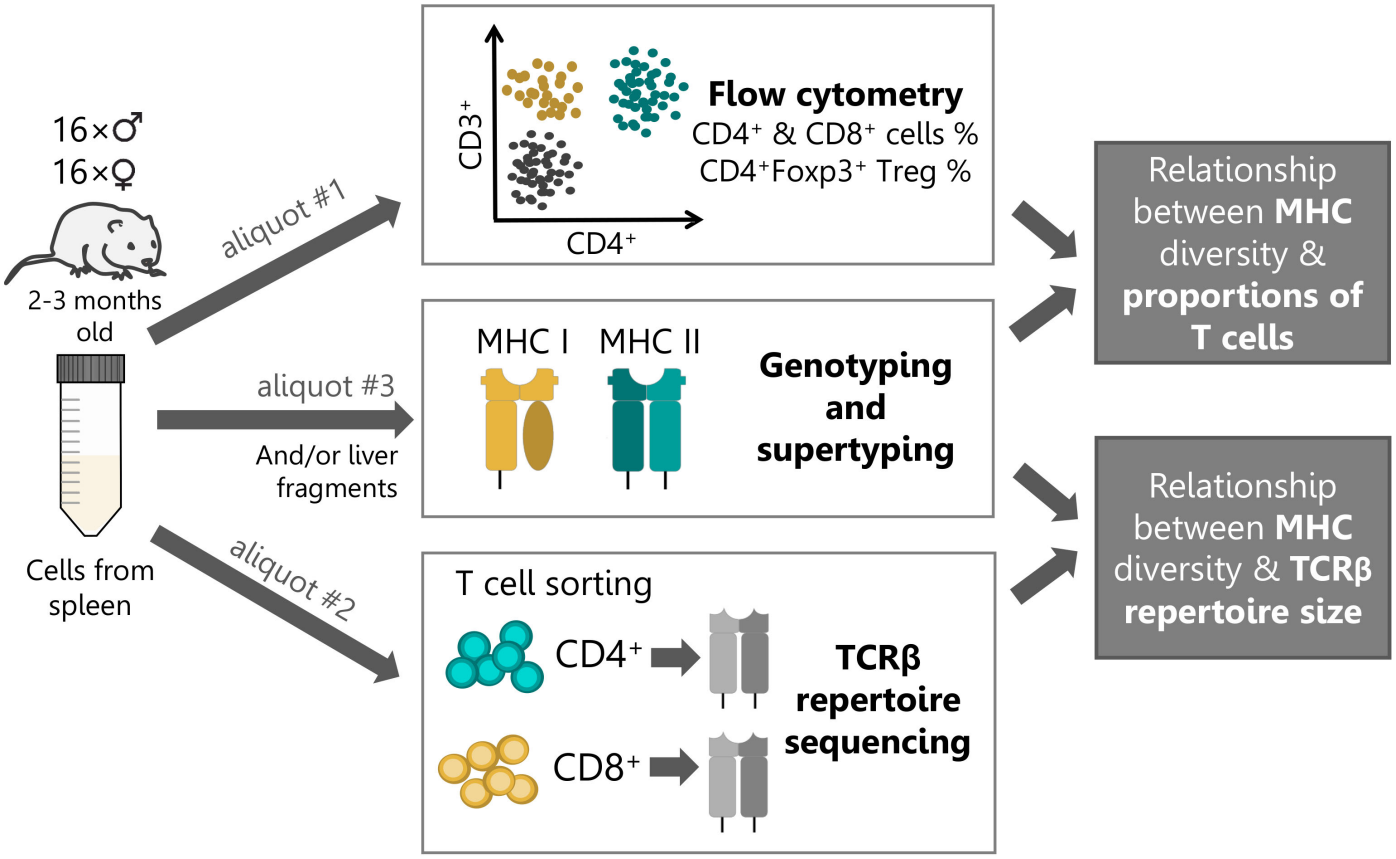
Experimental workflow. Single-cell suspension of splenic lymphocytes from each bank vole (n=32) were split into three aliquots and used in subsequent procedures: #1 – detail cytometric analysis; #2 - T cell sorting (10^5^ cells per subset) and subsequent high-throughput TCR repertoire sequencing; #3 - MHC genotyping by sequencing and supertyping. In addition, material from liver tissue was used for MHC genotyping, alongside the splenic material (or instead, if the amount of the former was insufficient).

In the current study, we set out to investigate how the number of expressed MHC variants in each class (that is, amino acid or supertype variants amplified across duplicated loci; a measure hereafter referred to as MHC diversity) influence splenic proportions and TCR repertoires of responding T cell subsets of bank voles (**Figure 1**). First, we examined how MHC class I diversity affects the proportion of cytotoxic CD8+ T cells, and how MHC class II affects the proportion of helper CD4+ and regulatory CD4+Foxp3+ T cell subsets. Second, we investigated how the diversity of a particular MHC class affects the size of TCR repertoire of the responding T cell subset. Studying these parameters sheds light on causes for the observed disparity between MHC class I and II, and informs our understanding of the trade-offs shaping the adaptive immunity of vertebrates.

## 2 Materials and methods

### 2.1 Animals

Young and healthy animals were obtained from a laboratory colony maintained at the Institute of Environmental Sciences, Jagiellonian University, Krakow (31), in accordance with resolution no 258/2017 of the 2nd Local Institutional Animal Care and Use Committee in Kraków. The laboratory colony was established from a large number of wild-caught individuals as an experimental evolutionary model with three directions of selection and unselected, control lines. Rearing procedures were designed to maintain genetic diversity [via e.g., avoiding sibling mating (31)] and a previous study that used animals from the same colony showed levels of MHC CNV similar to those seen in the wild (26). At the same time, standardized conditions and controlled environment minimized differential exposure to both abiotic factors and antigens (e.g., food or airborne, or coming from commensal microbiota) that might skew immune phenotypes. To further limit the source of variability, only animals from the control group (from 12 families representing all four control lines) were used in the current work.

### 2.2 Tissue processing

Thirty-two bank voles (16 males and 16 females) were processed in eight batches of four animals per day. We used 10–13 weeks old animals (sexually mature, young adults), culled in routine colony size maintenance procedures. Animals were sacrificed by cervical dislocation and organs were harvested immediately thereafter. Spleen harvest, preparation of single cell suspension and erythrocyte lysis were performed as described in Migalska et al. (32). Cells from each spleen were then counted and divided into three aliquots (**Figure 1**): #1) 1.5-2.5×10^6^ cells were used for staining and detailed flow cytometric analysis; #2) 6-10×10^6^ cells were used for cell staining and subsequent sorting into putative helper CD4^+^ and cytotoxic CD8^+^ T cell subsets; #3) remaining material (typically approx. 1×10^6^) was reserved for MHC genotyping. In addition, small liver fragments were collected from each individual at the time of necropsy and stored in RNAlater (Sigma-Aldrich).

### 2.3 Flow cytometry

A monoclonal antibody (mAb) cross-reactivity study reported in (32) showed that a combination of commercially available mAbs against CD4, CD3, and Foxp3 allows flow cytometric discrimination of the main subsets of T cells in bank voles, i.e., putative: helper CD4+, cytotoxic CD8+ (as CD3+CD4-), and regulatory CD4+Foxp3+, despite the lack of cross-reactive mAbs directly targeting CD8 molecules. The following mAbs were used: rat anti-mouse CD4 (clone GK1.5, eBioscience), rat anti-mouse Foxp3 (FJK-16s, Invitrogen) and rat anti-human CD3 (CD3-12, BioRad). In the current study, flow cytometry was used for a detailed inter-individual comparisons of T cell subset proportions in the spleen as well as for T cell sorting into CD4+ and CD8+ subsets (prior to TCR repertoire sequencing).

Cell staining for detailed cytometric analysis was performed on splenocyte aliquot #1 from the *Tissue processing* section, according to a protocol described in REF (32). (details also in the **Supplementary Materials**, **Supplementary Methods** section). For each individual, cell staining with mAbs was performed in three technical replicates (minimum 0.5×10^6^ cells/replicate). Cells were analyzed on a CytoFLEX cytometer (Beckman Coulter). The gating strategy is described in **Supplementary Methods**, **Supplementary Figure 1**. The proportions of specific T-cell subsets used in subsequent analyses were the mean of the three technical replicates.

Cell staining for sorting used splenocyte aliquot #2 (*Tissue processing* section) and followed a modified protocol of Channathodiyi & Houseley (33), that was developed to best preserve RNA quality during intracellular staining. Major modifications include the use of glyoxal to fix cells and addition of RNase inhibitors into the incubation and wash solutions. The exact protocol was described in REF. (32). Cells were sorted on FACS Aria IIIu (Becton Dickinson). 10^5^ of CD4+ (i.e., CD3+CD4+) and CD8+ (i.e., CD3+CD4-) cells were collected in PBS supplemented with RNasin® Plus RNase Inhibitor (Promega). After sorting, cells were centrifuged at 1800g for 3 minutes at 4°C, and the supernatant was removed. Cells were then lysed with 350μl of RLT buffer (Qiagen) and kept on ice until RNA extraction.

### 2.4 RNA extractions, amplicon library preparation and high-throughput sequencing (HTS)

#### 2.4.1 MHC

For each vole, RNA from two independent isolates was used for MHC genotyping. RNA was extracted from either one liver fragment and ≥1 × 10^6^ of splenocytes (i.e., aliquot #3 in the *Tissue processing* section) (n=19) or, if the amount of splenocytes was insufficient for a successful RNA extraction, from two liver fragments (n=13). These tissues were chosen because they contain relatively high amounts of MHC class II-expressing APCs (MHC class I is expressed ubiquitously) (34, 35). Total RNA was extracted with RNAzol, according to the manufacturer’s instructions (details in the electronic **Supplementary Material**, **Supplementary Methods**). The extracted RNA was reverse transcribed with oligo(dT) primers and the Maxima H Minus First-Strand cDNA Synthesis Kit (ThermoFisher Scientific, Waltham, MA, USA), according to the manufacturer’s protocol. Because MHC class II expression is much higher in spleen than in liver, we used 250-500ng of total RNA from splenocytes and 3-6μg of total RNA from livers for transcription (to normalize the amount of template for subsequent PCRs).

Four MHC gene fragments were amplified: third exon of MHC class I and second exons of MHC class II DQA, DQB, and DRB genes. Primers for amplification of these hypervariable exons encoding fragments of peptide-binding clefts of MHC molecules were taken from REFs (26, 27). Four independent PCR amplicons (two per RNA extraction) were generated for each gene, MHC amplification and library preparation was done as described in REF (26). (detailed description is also in **Supplementary Methods**). Pooled amplicons were sequenced on MiSeq (Illumina) using MiSeq Reagent kit v2 (paired-end, 2×250 cycles) in a single sequencing run.

#### 2.4.2 TCRs

RNA was extracted on the day of cell collection using the RNeasy Mini Kit (Qiagen), according to the manufacturer’s instructions (elution in 20μl of RNAse-free H_2_O), and stored at -80°C until further processing. Amplicon library preparation and TCR repertoire sequencing followed the quantitative analysis protocol of (36), with minor modifications (described briefly below and in details in **Supplementary Methods**). First, all RNA from each sample (i.e., from 10^5^ sorted CD4+ or CD8+ T cells) was reverse-transcribed by 5′RACE using SMARTScribe(TM) Reverse Transcriptase kit (TaKaRA) and custom template-switch oligos containing Unique Molecular Identifiers (UMIs, random DNA sequences that uniquely tag individual cDNA molecules). The total cDNA was then purified and used as a template in the first PCR (Q5® High-Fidelity 2X Master Mix, NEB, 23 cycles). After purification, 5μl of PCR 1 product was used as a template in the second PCR (Q5® High-Fidelity 2X Master Mix, 12 cycles). Products of the PCR 2 (i.e., uniquely tagged, finished libraries) were purified, pooled in equimolar quantities and sequenced by Novogene (Cambridge, United Kingdom) on NovaSeq Illumina (paired-end, 2×250-bp cycles) in two sequencing runs to reach depth of at least 3×10^5^ paired reads per sample (mean per sample: 5.22×10^5^, range: 2.98×10^5^ - 8.68×10^5^; **Supplementary Table 1**). An important change from the previously published protocol (36) was the sequencing of only one replicate per sample, instead of four. This change was made because the use of a standardized number of cells per assay prompted switch to abundance-based Chao1 estimator for repertoire analysis (see below) rather than incidence-based Chao2.

### 2.5 HTS data processing and analyses

#### 2.5.1 MHC genotyping and supertyping

MHC genotyping was performed as described in (26); details of the parameters used and modifications of the protocol can be found in the **Supplementary Methods**. Briefly, we used the “adjustable clustering” method (37) with AmpliSAS software (38) for demultiplexing, clustering artifacts with true variants and filtering of variants according to user-specified parameters. MHC variants were called in the individual genotypes if they were present in two out of four independent PCR replicates. The use of the amplification repeatability, rather than just read abundance, was motivated by the choice of RNA as the starting material (which may introduce additional, expression-level related variation in the read depth among alleles). Since in most cases we used RNA from two different tissues (i.e., liver and spleen), we refined our genotyping approach for MHC class I (details in **Supplementary Methods**) to sieve out potential non-classical MHC Ib genes. In contrast to classical, polymorphic and ubiquitously expressed MHC Ia molecules, MHC Ib are characterized by tissue-restricted expression patterns, low polymorphism and specialized functions other than presentation of peptides to cytotoxic CD8+ αβ T cells.

After genotyping, nucleotide sequences were trimmed to include only the focal exon, then translated into amino acid sequences, and variants encoding identical protein sequences were collapsed. Next, we assigned MHC variants to supertypes (clusters of variants grouped by the physicochemical properties of the peptide binding residues, PBRs) (39) using previously published positively selected sites (PSSs) in the MHC of this species (26–28, 40) as proxies for PBRs. Supertyping was performed as described in (26), details are also available in the **Supplementary Methods**. Finally, individual MHC diversity was summarized by providing the number of unique supertypes counted for the third exon of MHC class I (i.e., MHC class I diversity) and sum of unique supertypes counted for second exons of DQA, DQB and DRB (i.e., MHC class II diversity). Analogous counts were provided for numbers of amino acid variants.

#### 2.5.2 TCR repertoire analyses and size estimation

TCR amplicons were concatenated, filtered, error-corrected with UMIs and processed to extract TCRβ CDR3 region as described in (26). To standardize data input prior to subsequent analyses, we subsampled all amplicons to 3×10^5^ reads (the number at which Chao1 estimations leveled in rarefaction curves; see **Supplementary Figure 2B**, **Supplementary Methods** for details). For each sample that reached sufficient sequencing depth (i.e., 3×10^5^, n=27 for sorted CD4+ cells; n=28 for sorted CD8+ cells), we calculated the number of CDR3 sequences (i.e., the observed TCRβ repertoire, **Supplementary Table 1**). We also checked the overlap between the observed TCR repertoires within CD4+ and CD8+ subsets. Furthermore, to estimate the lower bound of the TCRβ richness, we used a non-parametric, abundance-based Chao1 estimator (41). This is a method developed to address the “unseen species” problem in ecological censuses, and is now routinely used to estimate the size of immune repertoires [e.g., (42, 43)]. Per-sample clonotype diversity and abundance data (following an UMI-based error correction), as well as between sample overlap, were obtained using the AmpliCDR3 tool (36) and analyzed in R (44) (v. 4.0.2).

### 2.6 Statistical analyses

We tested associations between i) MHC diversity and proportions of T cell subsets among all splenic lymphocytes; ii) MHC diversity and the size of TCR repertoires of corresponding T cell subsets. All the models were run with the numbers of MHC supertypes as explanatory variables, and repeated with the number of MHC amino acid variants. Model results were visualized with package effects (Effect Displays for Linear, Generalized Linear, and Other Models, v.4.2-2) (45, 46) or bayesplot (Plotting for Bayesian Models, v. 1.10.10) (47). All calculations were performed with R (44) (v. 4.0.2) in R Studio v.2022.12.0.

In the first set of analyses, proportions of T cell of each subset (quantified by flow cytometry) were compared to the individual MHC diversity. Given the nature of response variables and following the recommendations of Douma & Weedon (48), we chose beta regression (49) for statistical modeling. To that end, we used generalized mixed-effect models implemented in R package glmmTMB (50) (v. 1.1.7), with family = “beta_family” and link = “logit”. In all cases, the response variable was the proportion of T cells of a given subset, and the explanatory variable was the number of MHC variants of corresponding class. That is, the number of MHC class I variants was used to explain the proportion of CD8+ T cells in all lymphocytes; the number of MHC class II variants was used to explain the proportion of CD4+ T cells in all lymphocytes and of CD4+Foxp3+ T cells in either all CD4+ T cells or all lymphocytes. Additional explanatory variables used in each model were: sex (as a fixed effect); batch (see *Tissue processing* section) and family from which the animal came from (as random effects). Finally, we added a quadratic term to check for a possible non-linear relationship between the T cell proportions and MHC variant numbers, and we checked (using a likelihood ratio test, LRT) whether it improved the model fit. Conformation to model assumption and lack of overdispersion was confirmed with DHARMa R package (v. 0.4.6) (51).

Alternatively, rather than calculating separate models, the proportions of T cells among splenic lymphocytes could be treated as a single proportion with three categories, i.e., CD4+ T cells, CD8+ T cells and other lymphocytes (such as B cells). An extension of beta regression to cases when proportions are composed of more than two categories is provided by Dirichlet regression (52), currently implemented for mixed-effect models only within Bayesian framework. We used package brms (53), v 2.18.0, to run a model with family=Dirichlet, and the third category: other lymphocytes (i.e., CD4-CD3- cells) treated as a “reference category”, relative to which all coefficients were calculated. The same fixed and random effects were used as above, however here, effects of both MHC classes were estimated in one model. Normal prior distributions (μ=0, σ2 = 1) were used for fixed effects, and a package default (half Student-t prior with 3 degrees of freedom) for random effects. Models were run with four chains, 2000 iterations per chain (first 1000 were discarded as burn-in) and checked for convergence with Rê statistics and a visual inspection of chain traces.

In the second set of analyses, TCRβ Chao1 diversity estimates from a specified number of sorted T cells (i.e., 10^5^) were regressed on the individual diversity at a corresponding MHC class. To do so, we first fitted linear mixed-effect models with random effect structure as above, but they consistently yielded zero or near zero variance for the random effects and resulted in singular fits. Therefore, as these random effects were introduced to control for non-independence of observations, but their variability was not of scientific interest, we dropped them and used a simpler, linear model implemented in base R package stats. In these models we used sex and either MHC class I variant number to explain TCR repertoire of CD8+ T cells, or MHC class II variant numbers for the TCR repertoire of CD4+ cells. We also checked whether an addition of quadratic term for MHC gene diversity would be justified.

## 3 Results

### 3.1 MHC genotyping and supertyping

Majority of detected, expressed nucleotide MHC variants was previously described in the studied laboratory population of bank voles (26, 27); sequences of the new variants were deposited in Genbank (32 out of 83 detected for MHC class I; 6/31 for DQA, 7/38 DQB, 4/28 DRB). Average per individual number of MHC amino acid variants/supetypes was 12.4/9.8 in case of MHC class I and 11.2/10.1 for MHC class II (sum of DQA, DQB and DRB); detailed summary is in the **Supplementary Table 2**, final genotypes are in the **Supplementary Dataset 1**). There was no correlation between the number of variants/supertypes between the two classes (r_30_ = 0.098, p=0.59 for amino acid variants, r_30_ = 0.116, p=0.53 for supertypes).

### 3.2 T cell subsets and TCR repertoire estimations

Percentages of T cells among splenic lymphocytes varied between bank voles (**Supplementary Dataset 2**). On average, 27.9% (± SD: 5%, range: 18.8 -37.9%) of lymphocytes were identified as CD4+ T cells and 20.6% (± 4%, range: 10.5-27.7%) as CD8+ T cells. The remaining 51.4% (± 6.7% range: 39.5-66.3%) of splenic lymphocytes were CD3-CD4- cells (likely B cells). The proportions of CD4+ and CD8 T cells were not correlated (r_30_ = 0.119, p=0.52). Mean CD4/CD8 ratio was 1.4 ( ± 0.4, range: 0.8-2.2). There was a tendency for lower CD4/CD8 ratio in males than in females (mean values 1.3 and 1.5, respectively), but the t-test was marginally non-significant (p value = 0.069, t = 1.88, df = 30). Within CD4+ T cell subset, on average 8.4% (± 1.7%, range: 5.7-13.5%) were putative regulatory T cells (Treg; i.e., CD3+CD4+Foxp3+). Moreover, there was a negative correlation between the proportion of CD4+Foxp3+ in CD4+ T cells and the overall proportion of CD4+ T cells in spleen (r_30_= -0.4, p=0.025).

Observed clonotype richness (i.e., number of unique, nucleotide CDR3 sequences) in 10^5^ sorted T cells ranged between 2.8-13.3×10^3^ (on average 7.9×10^3^) in CD4+ T cells and between 2.7-14.4×10^3^ (on average 6.8×10^3^) in CD8+ T cells (**Supplementary Table 1**). The clonal proportion analysis did not showed major expansions. Ten top most abundant clonotypes occupied on average 2.4% (max: 8.7%) of the observed repertoire, first 1000 most abundant clones occupied on average 30% of the repertoire space (**Supplementary Figure 3**). Within-individual overlap between the observed TCR repertoires of CD4+ and CD8+ subsets (n=27) was low, on average 1% (0.5-1.6%) for nucleotide CDR3 sequences, and higher for translated amino acid CDR3s (mean 4.2%, 1.8-6.3%). Lower bound Chao1 TCR repertoire size estimates ranged between 1.0-5.5×10^4^ (on average 3.1×10^4^) in CD4+ T cells and between 1.2-4.3×10^4^ (on average 2.7×10^4^) in CD8+ T cells (**Supplementary Table 1**). The estimated TCR richness based on 10^5^ sorted cells of that subset did not significantly correlate with the proportion of T cells of a given subset (r_27_ = 0.15, p=0.486 for CD4+ T cells, r_25_ = 0.02, p=0.904 for CD8+ T cells). Instead, individual TCR repertoire richness estimates for these subsets were strongly, positively correlated with each other (r_25_ = 0.75, p<0.001, **Supplementary Figure 4**). At the same time, overall TCR richness did not correlate with differences in either cell viability or total splenic cell counts (see **Supplementary Methods** for details).

### 3.3 Relationship between MHC diversity and proportions of T cells

**Figure 2:**
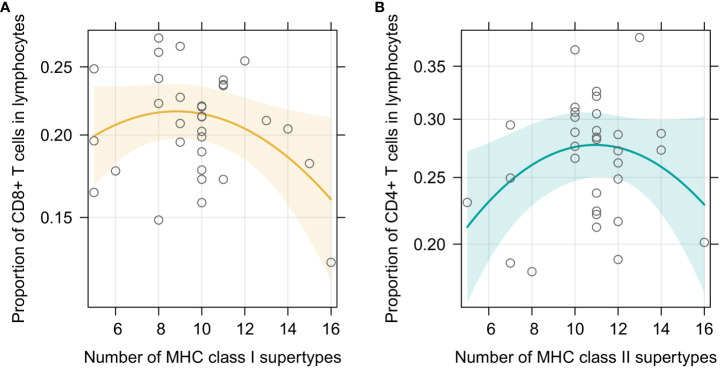
Predictor effect plots of **(A)** number of MHC class I supertypes on the proportion of CD8+T cells among splenic lymphocytes; **(B)** number of MHC class II supertypes on the proportion of CD4+T cells among splenic lymphocytes. Line shows fitted values versus the focal predictor on the horizontal axis, when other predictors are held fixed. The shaded area is a pointwise confidence band for the fitted values, based on standard errors computed from the covariance matrix of the fitted regression coefficients. Hollow points are partial residuals.

In the first set of models we checked how our focal predictors, i.e., sex and individual MHC diversity, affected characteristic of the splenic lymphocyte landscape. In the beta regression GLMMs, proportion of CD8+ T cells was marginally affected by sex, with a tendency to be higher in males (p=0.092). Moreover, it was affected by MHC class I diversity, but in a non-linear manner (**Figure 2A**) – individuals with an intermediate number of MHC supertypes had the highest proportion of CD8+ T cells (p= 0.048 for the quadratic term; full model results are in **Supplementary Table 3**). Model with a quadratic term fitted better than the one without it (df=1; χ^2^ = 4.012; p=0.045). Model with MHC amino acid variants yielded similar results (**Supplementary Figure 5A**, **Supplementary Table 4**). Proportion of CD4+ T cells was similarly affected by the number of MHC class II supertypes (p= 0.035 for the quadratic term, **Figure 2B**, **Supplementary Table 5**), but there was no effect of sex (p=0.346). Addition of the quadratic term improved the model fit (df=1; χ^2^ = 4.012; p=0.045). In a model with the amino acid MHC class II variants this relationship was also present, but was only marginally significant (**Supplementary Figure 5B**, **Supplementary Table 6**). None of our focal explanatory variables predicted the proportion of CD4+Foxp3+ T cells within CD4+ T cells in the bank vole spleens, i.e., there was no significant effect of sex or the numbers of MHC class II supertypes/amino acid variants (**Supplementary Tables 7, 8**). Given the negative correlation between proportion of CD4+Foxp3+ T cells within CD4+ T cell subsets and total CD4+ levels in spleen, we added the proportion of CD4+ T cells as an explanatory variable in the model. It improved the fit (df=1; χ^2^ = 8,175; p=0.004) and eventually proved to be the only significant predictor of the proportion of CD4+Foxp3+ T cells within CD4+ T cells (coeff. = -2.144, p= 0.002, **Supplementary Figure 6A**, **Supplementary Tables 9, 10**). This suggested that the proportion of regulatory CD4+Foxp3+ T cells among T cells might be rather constant and fairly independent from the overall proportion of CD4+ T cells. Then we performed an analysis aimed at explaining the proportion of CD4+Foxp3+ T cells in all lymphocytes (**Supplementary Figure 7**), rather than just in CD4+ T cells. It showed no effect of sex (p=0.671), but revealed a non-linear relationship between the number of MHC variants and cells proportions (p=0.006 for quadratic term, addition of term was supported by a LRT: df=1; χ^2^ = 7,328; p=0.007; **Supplementary Figure 6B**, **Supplementary Table 11**), similar to the one reported above. Analysis with the numbers of MHC class II amino acid variants yielded equivalent results (**Supplementary Table 12**).

**Figure 3:**
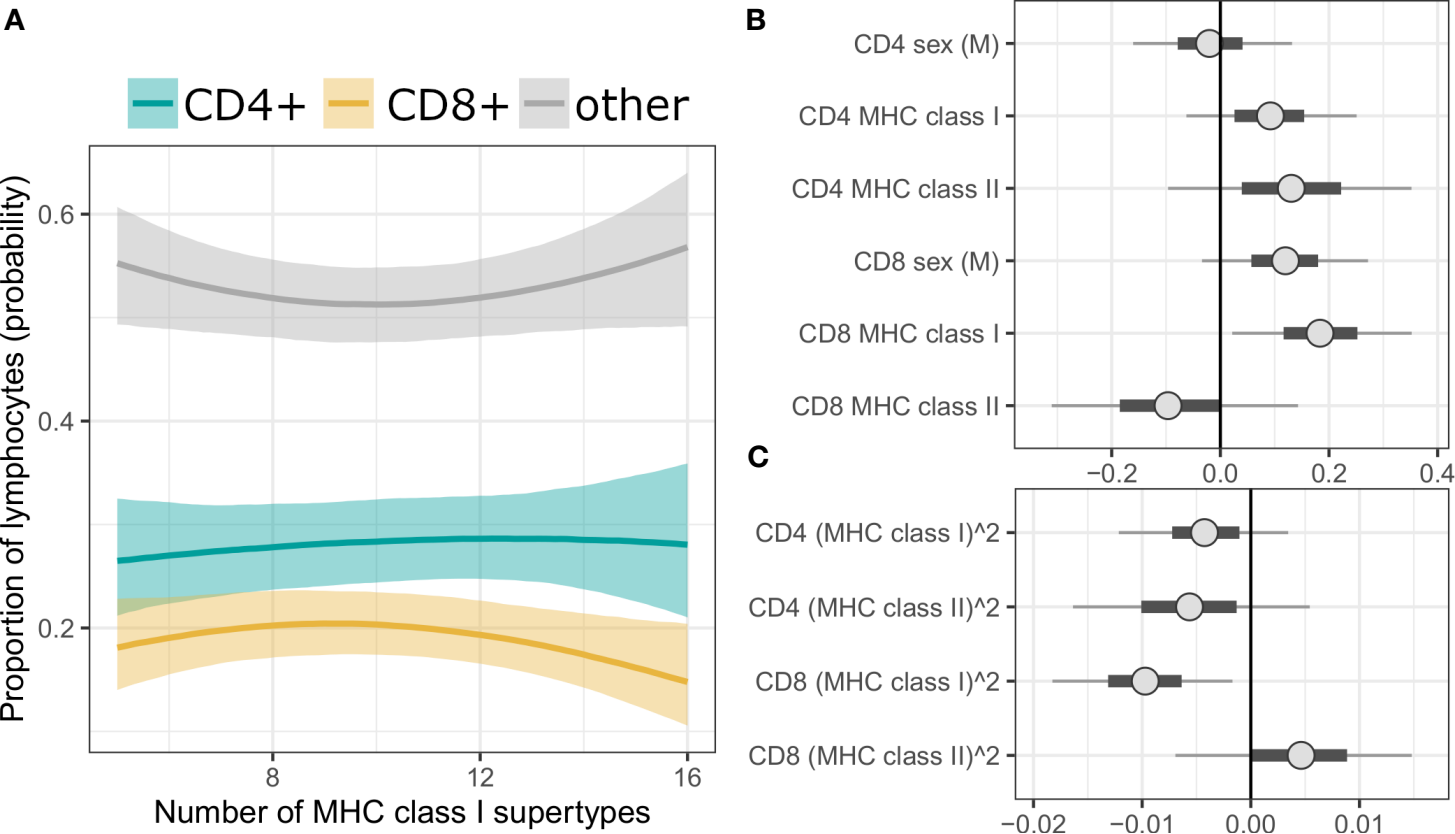
**(A)** Conditional effect plot of MHC class I supertype number on splenic lymphocyte proportions. The three response categories are proportion of CD4+ T cells (teal line), CD8+ T cells (yellow line) and other splenic lymphocytes (grey line). Shaded are shows 90% credible intervals (CI). **(B, C)** Posterior CI visualizing Markov chain Monte Carlo draws from the posterior distribution of the parameters of a Bayesian model with MHC supertypes and sex as predictors. “CD4” or “CD8” in the parameter prefixes corresponds to the predicted effect on given T cells subpopulation (third, reference category, i.e., “other lymphocytes”, was modeled implicitly). Figure was split into panel **(B)** (linear term) and **(C)** (quadratic term) to better visualize posterior values at different scales. The points are posterior medians, thick segments are 50% CI, thinner lines are 90% CI (default).

We also performed a complementary, Bayesian analysis with Dirichlet regression, where the proportions of T cells among splenic lymphocytes was treated as a compositional proportion with three categories (i.e., CD3+CD4+ as CD4+ T cells; CD3+CD4- as CD8+ T cells; CD3-CD4- as other lymphocytes). It revealed similar tendencies as described above, however results were statistically robust only for the prediction of CD8+ T cell proportion by MHC class I, i.e., the 95% credibility intervals (CI) of the posterior parameter estimates did not span over zero (**Figure 3** for MHC supertypes, **Supplementary Figure 8** for amino acid variants, **Supplementary Tables 13, 14** for full models).

### 3.4 Relationship between MHC diversity and TCR repertoire size

In the second set of models, we checked how MHC diversity affected the TCRβ repertoire size of the T cell subset interacting with a given MHC class. To decouple the TCR diversity from the differences in cell proportions, we used a uniform number of sorted cells from each subset (i.e., 100 000) for TCR sequencing. Furthermore, we used Chao1 richness estimator to calculate (based on the observed abundance of CDR3 clonotypes) a lower bound of the TCR diversity (hereafter “TCR richness”). We did so to account for inevitable cell loss during laboratory procedures and general technical limitation associated with HTS immune repertoire analysis.

**Figure 4:**
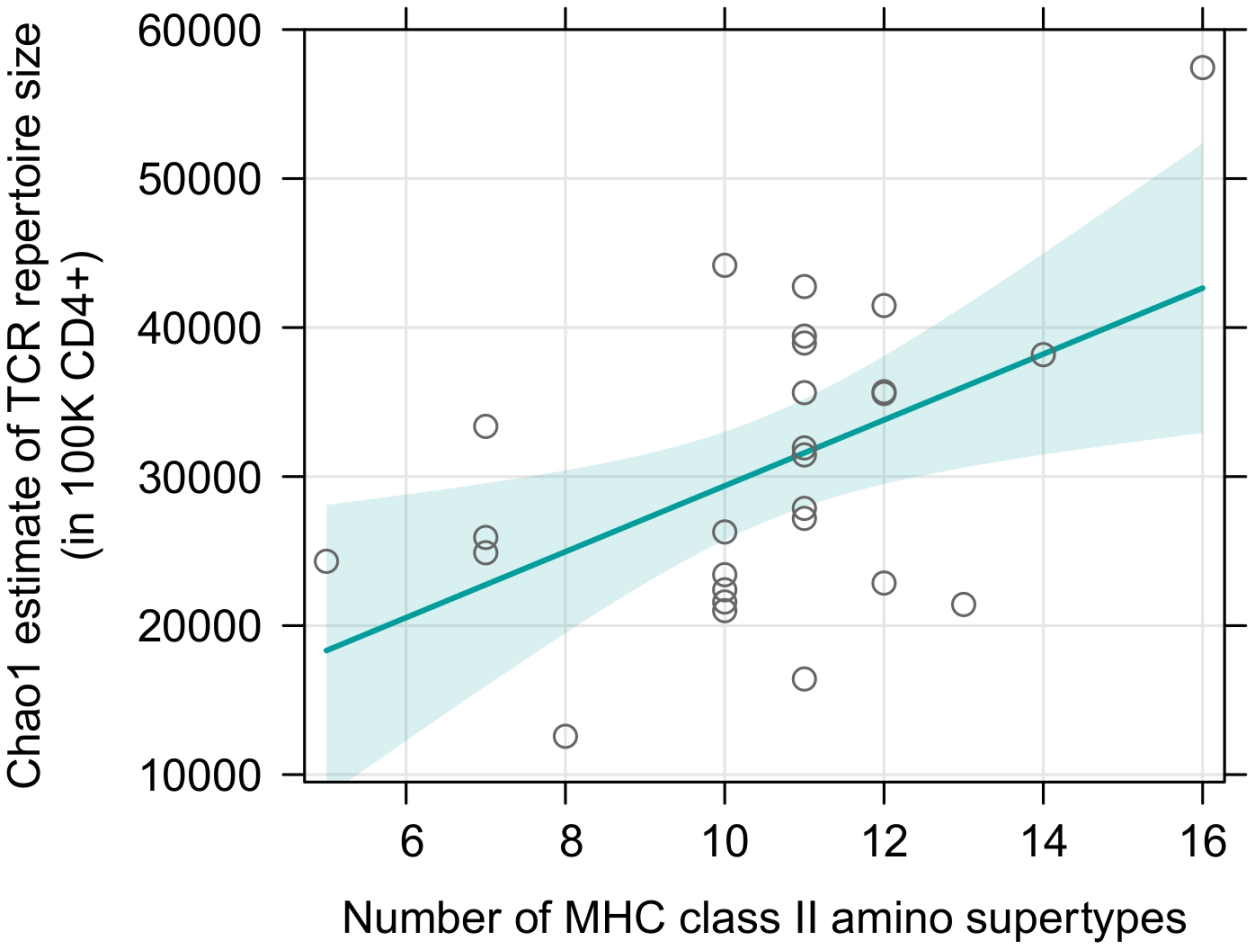
Predictor effect plots of number of MHC class II supertypes on the Chao1 TCR diversity estimates in 10^5^ sorted CD4+ T cells. Teal line shows fitted values versus the focal predictor on the horizontal axis, when other predictor (sex) was held fixed. The shaded area is a pointwise confidence band for the fitted values, based on standard errors computed from the covariance matrix of the fitted regression coefficients. Hollow points are partial residuals. For an equivalent figure, without the influential observation – see **Supplementary Figure 10**.

Surprisingly, there was no effect of either sex or MHC class I diversity on the TCR richness of sorted CD8+ T cells and this result was consistently obtained for both supertype and amino acid variants diversity (**Supplementary Tables 15, 16**). Addition of a quadratic term did not improve the fit (df=1; F= 0.024; p=0.879 for supertypes; F= 0.261; p=0.613 for amino acid variants). In contrast, MHC class II diversity was positively correlated with the TCR richness of sorted CD4+ T cells (p=0.011, **Figure 4**, **Supplementary Table 17**); the quadratic term was omitted, as its addition was not supported: df=1; F= 3.264; p=0.084). The relationship, however, seemed to be driven mostly by one datapoint, namely an individual with a large number of MHC class II supertypes (16) and a large CD4+ T cell TCR diversity (Chao1 estimate >5.5×10^4^). Indeed, Cook’s distance measurement for this observation was 0.45 (**Supplementary Figure 9**), proving it was an influential point in this analysis (i.e., it was higher than a conservative rule of thumb which sets a threshold at 4/n; in this case 4/27 = 0.15). However, careful analysis of the data did not reveal any suspicious characteristics that would indicate that any of the measurements for this individual was artificially inflated due to a technical error, therefore we do not believe it should be removed from the analysis. Nonetheless, we also repeated the test without this observation, which rendered the trend non-significant (p=0.105, **Supplementary Figure 10**, **Supplementary Table 18**). Models with number of amino acid variants, instead of supertypes, yielded similar results (**Supplementary Figure 11**, **Supplementary Tables 19, S20**).

## 4 Discussion

One of the evolutionary puzzles lurking in the vertebrate adaptive immunity is the question why a population-level advantage of a huge allelic MHC polymorphism does not translate into an accumulation of MHC loci in genomes, yielding similar benefits at the individual level. One favored explanation, the mathematically formalized T cell repertoire depletion hypothesis (6, 7), posits that gains in pathogen recognition obtained by an individual through additional MHC alleles would be offset by an excessive depletion of T cells bearing self-reactive TCRs during thymic selection. While allelic polymorphism of the MHC is known to affect the composition of the T cell compartment and the TCR repertoire (43, 54–56), proximal effects of MHC gene duplication are far less understood. Moreover, a recent, overall test of the T cell repertoire depletion hypothesis by Migalska et al. (26) have raised the idea that the two MHC classes may enact the proposed evolutionary trade-offs differently. Here, we go a step further and examine how diversities of the two MHC classes influence both the proportions and the TCR repertoire sizes of the major, functional T cell subsets in a species with an extensive CNV in MHC.

We found that an intermediate number of expressed MHC supertypes or amino acid variants (hereafter: MHC diversity) predicted highest proportions of responding T cell subsets. This non-linear relationships may reflect another facet of the MHC optimality, that could result from balanced efficiencies of the thymic selection steps. In its original formulation, the optimality effect (predicted for parasite load/diversity in individuals with intermediate numbers of MHC genes) was assumed to result from, on one end, poor antigen presentation by individuals with too few MHC alleles, and on the other end, TCR repertoire depletion in individuals with too many MHC alleles. Our results suggest that costs at both extremes may be mediated by suboptimal T cell numbers, whereas an intermediate MHC diversity may promote robust T cell survival during positive selection, but not yet reach levels that cause an exaggerated negative selection. If confirmed, this model could finally reconcile conflicting arguments emphasizing the role of either positive (10) or negative (6) selection in the MHC gene number evolution. We also note the limitations of our current approach. Primarily, the lack of antibodies specifically recognizing the CD8 molecule in bank voles precluded a more direct estimation of the role of the thymic selection by inspecting the proportions of single positive T cells in the thymus itself. There, the presence of double positive CD4+CD8+ cells prevented the strategy used in the spleen, i.e., the use of anti-CD4 and anti-CD3 mAbs to identify CD4+ and CD8+ T cells (as CD3+CD4+ and CD3+CD4- subsets, respectively). Examination of other lymphoid tissues, e.g., lymph nodes, might also provide a somewhat different picture, but the current experimental design (**Figure 1**) required cell yields that were readily achievable only from the spleen. Finally, the limited availability of molecular reagents precluded the identification and selection of only naïve T cells for our analyses. Nevertheless, the impact of an individual MHC diversity seemed to pervade into the periphery, allowing the detection of its signatures also in the spleen, where e.g., a homeostatic proliferation of T cells or expansion upon contact with certain antigens present in the colony could obscure the signal.

The lack of effect of MHC class I on CD8+ T cell TCR richness and a positive correlation between MHC class II and CD4+ TCR richness were surprising, especially in the light of recent findings in the bank vole (26) and in humans (57). Previously, a negative correlation between the MHC class I diversity and the TCRβ repertoire size was reported in the bank vole (26), whereas the opposite trend was found in humans (57). In contrast, both studies found no correlation between MHC class II and TCRβ repertoire diversities (26, 57). However, several important differences in both the study systems and/or design may have contributed to these inconsistencies, and when carefully considered, may be reconciled with our current findings.

First, both previously reported TCRβ repertoire diversity estimates were based on bulk TCR sequencing of either aliquots of RNA extracted from whole spleens [in bank voles (26)] or genomic DNA extracted from blood mononuclear cells, PBMCs [in humans (57)]. The diversity of TCR repertoire obtained in this way not only combined the contribution of both CD4+ and CD8+ T cells, but was also influenced by varying proportions of these cells. This “composite” measure cannot be easily compared to the TCR richness of sorted, uniform cell numbers of the two subset studied here.

Second, our current study differs from the previous reports in both the extent and the distribution of the MHC diversity, which has important implications for the analysis and interpretation of the results. In the case of the bank vole study, the TCR repertoire was sequenced for a pre-selected set of bank voles expressing ether a low or high number of MHC genes (both classes combined), leaving gaps in the middle of the MHC gene number distribution that discouraged fitting non-linear relationships (26). The current collection of animals reflects a more typical for the species, bell-shaped distribution of the MHC diversity. In the case of humans, especially for the MHC class I, the natural range of the individual MHC class I diversity does not overlap with that of bank voles (i.e., in humans there were between three and six alleles across three HLA-I loci, whereas in the bank vole, we observed between five and 16 MHC class I variants).

Previously observed, negative associations between MHC class I and TCR diversity in the bank vole are consistent with the fact that while the effect of the MHC diversity on T cell proportions shown here was visible for both MHC classes, it was robustly supported only for the MHC class I and CD8+ T cells. It is in agreement with a suggestion that a strong influence of the MHC class I genotype on the CD8+ lymphocytes is a general feature, resulting from a closer contact between TCRs of CD8+ cells and MHC I molecules, compared to those of CD4+ T cells and MHC class II (58). Moreover, the peak of the predicted relationship between MHC class I diversity and the proportion of CD8+ T cells was shifted toward lower numbers (from the central value of the observed range of MHC supertype numbers - 10.5, and from an observed mean value - 9.8, to approximately 8-9 variants, **Figures 2A**, **3A**). Given the lack of association observed here between MHC class I diversity and CD8+ TCR richness, if the effect on T cell proportions was dominant, it might have translated into a negative relationship between MHC class I diversity and the total splenic TCR repertoire size, as previously observed in the bank vole (26). In addition, if the overall relationship between MHC class I diversity and the total TCR repertoires was non-linear, the slope of the observed relationship will vary at different ranges of the MHC diversity. Thus, it is possible, that within the range observed in humans, increasing the MHC class I diversity up to six alleles still have a positive effect, whereas the situation in the bank vole reflects a diminishing (and eventually detrimental) effect of a high, individual MHC class I diversity on TCR repertoires.

If the MHC class II diversity affected CD4+ T cell proportions in a similar, non-linear manner to MHC class I, but increased the CD4+ TCR richness, the overall effect of MHC class II gene numbers on the total splenic TCR diversity may cancelled out. Thus, in a bulk sequencing setup (previously reported in the bank vole, 26), it might have appeared as if the MHC class II diversity did not affect the overall splenic TCR repertoire size. A similar mechanism could explain the lack of relationship between MHC class II allelic diversity and TCR repertoire richness from bulk PBMC described in humans (57). Notably, however, here we provide another line of evidence against the TCR depletion caused by a large, individual MHC class II diversity. It stands to reason that other evolutionary trade-offs may be thus key in limiting the number of MHC class II loci in genomes. One postulated mechanism is susceptibility to autoimmune disorders, which could result from insufficient elimination of self-reactive T cells during negative thymic selection and failure to establish self-tolerance (7). Equally, expansion of MHC class II loci could simply increase the likelihood of harboring MHC alleles associated with autoimmune disorders [the existence of which has been abundantly described, for example in humans (59, 60)] and promote further, non-additive, detrimental associations in autoimmune diseases (61).

In the previous article, Migalska et al. (26) hypothesized that the apparent lack of negative effect of MHC class II diversity on the overall TCR repertoire could be related to a covert expansion of a Treg subset, that while typically constituting <10% of CD4+ T cells, is characterized by a diverse and a largely distinct TCR repertoire (62). However, in the present study, we found no effect of MHC class II diversity on the proportion of Foxp3+ cells among splenic CD4+ T cells. This argues against the possibility that an increased MHC diversity would promote an expansion of the regulatory T cell subset within the CD4+ compartment. Instead, there appears to be a non-linear relationship between MHC class II gene number and the proportion of CD4+Foxp3+ T cells among all splenic lymphocytes, mirroring the general effect of MHC class II on CD4+ T cells. It is important to note, however, that we examined the Treg fraction in spleen, which includes both natural and induced Foxp3+ Tregs. The latter develop from naïve, conventional CD4+ T cells outside of the thymus, particularly at the mucosal interfaces of the gastrointestinal and respiratory tracts, and contribute to a tolerance to e.g., commensal microbiota antigens (63). We still cannot exclude the possibility that higher MHC class II gene numbers affect the generation of natural Tregs in the thymus, but subsequently cell proportions are normalized in the periphery. Indeed, a study investigating an influence of MHC class II allelic variation on T cell differentiation and TCR repertoire showed that in mice carrying an MHC haplotype associated with a deficiency of both conventional and regulatory CD4+ T cells in the thymus, the Treg population was later restored in the periphery (43).

Finally, we again acknowledge the technical limitations imposed by the limited array of mAbs available for our non-model rodent, which precluded sampling of only naïve T cells. Inclusion of some expanded, antigen-experienced T cells in the sorted compartments could affect the precision of estimating the TCR repertoire from a sample of 10^5^ cells and weaken the putative relationship with MHC diversity. However, although the bank voles did not come from a germ-free colony, the controlled and standardized environment made differential antigen exposure unlikely. Hence, given exposure to the same antigens, the observed differences in the TCR repertoires are likely related to the genetic makeup of the outbred individuals, of which MHC diversity is an important component, and thus these differences should still be interpretable in the light of the original hypothesis.

Another factor previously shown to influence the overall, splenic TCR repertoire of bank voles was sex, with males having a significantly smaller TCR repertoire than females (26). To follow up on these findings, in the current design, we matched the number of males and females and included sex as a predictor in all the statistical models. However, we found little support for an effect of sex on the measured parameters, except for some (mostly marginally non-significant) trends consistent with general immunological knowledge from model organisms, i.e., a tendency for a higher proportion of CD8+ cells and a lower CD4/CD8 ratio in males (64, 65).

Apart from the clear immuno-evolutionary focus, our study provided the first detailed characterization of the splenic lymphocyte landscape (supported by immune phenotyping with monoclonal antibodies, rather than transcriptomic data) in the bank vole, an emerging model species in ecology and evolution (31, 66–68). It is also a reservoir of pathogens with zoonotic potential, such as Puumala virus (69) or tick-borne bacterium *Borrelia afzelii* (70), which makes it a species of interest for parasitological and epidemiological surveillance (71, 72). We thus add bank voles to a short list of non-murine rodents (e.g., 73, 74) for which such immunophenotyping has been performed. Overall, the splenic frequencies of putative helper CD4+ and cytotoxic CD8+ T cells were similar to those reported in mice (75) and other Cricetidae rodents studied to date, such as the cotton rat (*Sigmodon hispidus*) (74). A predominance of CD4+ over CD8+ T cells is also typical, and has been described for several mouse strains (76), rats (77) and humans alike (64). The observed mean, splenic CD4/CD8 ratio of 1.4 was within the range reported for several inbreed or congenic mouse strains (43, 76) and was very similar to an indirect qPCR estimate reported previously in the bank vole itself [i.e., 1.5 ± 1.1 (26)]. The overall characteristics of the bank vole TCRβ repertoire have been described elsewhere (36); here we extended them to distinguish the repertoires of the CD4+ and CD8+ subsets. Similar to data reported in humans (78), we found very little intra-individual sequence overlap between the two subsets. TCR richness estimates were comparable to those obtained in mice in a similar experimental setup [i.e., number of sorted cells, diversity estimator used (43)]. Notably, the estimated TCR richness did not correlate with the observed proportion of T cells of the given subset (as quantified in the spleens), but was positively correlated between the two subsets. That is, the higher the TCR richness of CD4+ T cells, the higher the TCR richness of CD8+ T cell. It appeared to be a genuine phenomenon, rather than a technical or sampling artifact (i.e., it could not be attributed to differences in cell viability or total lymphocyte counts recorded for these individuals). A similar correlation has also been observed in e.g., humans (78).

## 5 Conclusions

Our study of the MHC class-specific consequences of thymic selection in a species with a variable number of MHC loci suggests a non-linear relationship between MHC diversity and T cell proportions, perhaps reflecting an optimality effect of balanced positive and negative selection. This association was most evident for the relationship between MHC class I and peripheral CD8+ T cells. The CD8+ TCR richness alone (decoupled from the T cell proportions) was unaffected by MHC class I diversity, suggesting that potential deleterious consequences of MHC class I expansion may be driven by decreasing T cell numbers (which may indirectly affect the TCR repertoire size), rather than by directly depleting the TCR richness of the responding T cell subset. In contrast, a positive correlation between the number of MHC class II variants and the diversity of the CD4+ TCRs argues against a universal TCR depletion model and calls for a revision of this long-standing hypothesis. It also suggests that an entirely different evolutionary trade-off may be instrumental in limiting the MHC class II expansion, such as susceptibility to autoimmune disorders. We hope that by highlighting these contrasting patterns and identifying limitations of the current approach, our work will inspire further, detailed investigations into the fascinating, yet understudied topic of the within-individual MHC diversity optima evolution.

## Data availability statement

New MHC variants are deposited in GenBank (OR424419-OR424467), raw sequencing files (for MHC and TCRs) are deposited in NCBI Sequence Read Archive, Bioproject: PRJNA1003007. Final MHC genotypes are provided in the **Supplementary Dataset 1**, associated intermediate genotyping files are in Open Science Framework (OSF) repository: https://osf.io/9yhfd/. Mean flow cytometric measurements and additional information on individuals (e.g., sex, exact age at death, processing batch, family designation in the colony etc.) are provided in the **Supplementary Dataset 2**, raw cytometric measurements (i.e., three technical replicates) are provided in OSF repository: https://osf.io/6wjrb/. Other **Supplementary Information** (**Supplementary Methods, Figures, Tables**) are provided in the on-line **Supplementary Materials File**.

## Ethics statement

Ethical approval was not required for the study involving animals in accordance with the local legislation and institutional requirements because animals used in this work were culled in routine size maintenance procedures at the bank vole colony held at the Institute of Environmental Sciences, Jagiellonian University, Krakow, Poland, in accordance with resolution no. 258/2017 of the 2nd Local Institutional Animal Care and Use Committee in Krakow. Therefore, no additional approval above what was already granted for the colony maintenance itself was needed.

## Author contributions

MM conceived and designed the study, performed bioinformatical and statistical analyses, wrote and revised the manuscript draft. MM, KW, KD, and JH performed laboratory procedures and/or data acquisition, JH contributed to the analysis of flow cytometric data. All authors contributed to the article and approved the submitted version.

## References

[1] Owen JA Punt J Stranford SA Jones PP. Kuby Immunology. 7th ed Vol. 832. . New York: W. H. Freeman and Company (2013).

[2] Spurgin LG Richardson DS. How pathogens drive genetic diversity: MHC, mechanisms and misunderstandings. Proc Biol Sci (2010) 277:979–88. doi: 10.1098/rspb.2009.2084 PMC2842774 20071384

[3] Radwan J Babik W Kaufman J Lenz TL Winternitz J. Advances in the evolutionary understanding of MHC polymorphism. Trends Genet (2020) 36:298–311. doi: 10.1016/j.tig.2020.01.008 32044115

[4] Doherty PC Zinkernagel RM. Enhanced immunological surveillance in mice heterozygous at the H-2 gene complex. Nature (1975) 256:50–2. doi: 10.1038/256050a0 1079575

[5] Vidović D Matzinger P. Unresponsiveness to a foreign antigen can be caused by self-tolerance. Nature (1988) 336:222–5. doi: 10.1038/336222a0 3143074

[6] Nowak MA Tarczy-Hornoch K Austyn JM. The optimal number of major histocompatibility complex molecules in an individual. Proc Natl Acad Sci (1992) 89:10896–9. doi: 10.1073/pnas.89.22.10896 PMC50449 1438295

[7] Woelfing B Traulsen A Milinski M Boehm T. Does intra-individual major histocompatibility complex diversity keep a golden mean? Philos Trans R Soc Lond B Biol Sci (2009) 364:117–28. doi: 10.1098/rstb.2008.0174 PMC2666699 18926972

[8] Cooper MD Alder MN. The evolution of adaptive immune systems. Cell (2006) 124:815–22. doi: 10.1016/j.cell.2006.02.001 16497590

[9] Klein L Kyewski B Allen PM Hogquist KA. Positive and negative selection of the T cell repertoire: what thymocytes see (and don’t see). Nat Rev Immunol (2014) 14:377–91. doi: 10.1038/nri3667 PMC4757912 24830344

[10] Borghans JAM Noest AJ De Boer RJ. Thymic selection does not limit the individual MHC diversity. Eur J Immunol (2003) 33:3353–8. doi: 10.1002/eji.200324365 14635043

[11] Yates AJ. Theories and quantification of thymic selection. Front Immunol (2014) 5:13. doi: 10.3389/fimmu.2014.00013 24550908 PMC3912788

[12] La Gruta NL Gras S Daley SR Thomas PG Rossjohn J. Understanding the drivers of MHC restriction of T cell receptors. Nat Rev Immunol (2018) 18:467–78. doi: 10.1038/s41577-018-0007-5 29636542

[13] Holland SJ Bartok I Attaf M Genolet R Luescher IF Kotsiou E. The T-cell receptor is not hardwired to engage MHC ligands. Proc Natl Acad Sci U.S.A. (2012) 109:E3111–8. doi: 10.1073/pnas.1210882109 PMC3494948 23077253

[14] Scott-Browne JP White J Kappler JW Gapin L Marrack P. Germline-encoded amino acids in the αβ T-cell receptor control thymic selection. Nature (2009) 458:1043–6. doi: 10.1038/nature07812 PMC2679808 19262510

[15] Baker BM Evavold BD. MHC bias by T cell receptors: genetic evidence for MHC and TCR coevolution. Trends Immunol (2017) 38:2–4. doi: 10.1016/j.it.2016.11.003 27939452 PMC5208042

[16] Feng D Bond CJ Ely LK Maynard J Garcia KC. Structural evidence for a germline-encoded T cell receptor–major histocompatibility complex interaction “codon”. Nat Immunol (2007) 8:975–83. doi: 10.1038/ni1502 17694060

[17] Scott-Browne JP Crawford F Young MH Kappler JW Marrack P Gapin L. Evolutionarily conserved features contribute to αβ T cell receptor specificity. Immunity (2011) 35:526–35. doi: 10.1016/j.immuni.2011.09.005 PMC3245739 21962492

[18] Harsha Krovi S Kappler JW Marrack P Gapin L. Inherent reactivity of unselected TCR repertoires to peptide-MHC molecules. Proc Natl Acad Sci U.S.A. (2019) 116:22252–61. doi: 10.1073/pnas.1909504116 PMC6825295 31570608

[19] Jerne NK. The somatic generation of immune recognition. Eur J Immunol (1971) 1:1–9. doi: 10.1002/eji.1830010102 14978855

[20] Van Laethem F Tikhonova AN Singer A. MHC restriction is imposed on a diverse T cell receptor repertoire by CD4 and CD8 co-receptors during thymic selection. Trends Immunol (2012) 33:437–41. doi: 10.1016/j.it.2012.05.006 PMC3427466 22771139

[21] Wegner KM Kalbe M Kurtz J Reusch TBH Milinski M. Parasite selection for immunogenetic optimality. Science (2003) 301:1343. doi: 10.1126/science.1088293 12958352

[22] Madsen T Ujvardi B. MHC class I variation associates with parasite resistance and longevity in tropical pythons. J Evol Biol (2006) 19:1973–8. doi: 10.1111/j.1420-9101.2006.01158.x 17040395

[23] Kalbe M Eizaguirre C Dankert I Reusch TBH Sommerfeld RD Wegner KM. Lifetime reproductive success is maximized with optimal major histocompatibility complex diversity. Proc Biol Sci (2009) 276:925–34. doi: 10.1098/rspb.2008.1466 PMC2664370 19033141

[24] Radwan J Zagalska-Neubauer M Cichoń M Sendecka J Kulma K Gustafsson L. MHC diversity, malaria and lifetime reproductive success in collared flycatchers. Mol Ecol (2012) 21:2469–79. doi: 10.1111/j.1365-294X.2012.05547.x 22512812

[25] Pikus E Dunn PO Minias P. High MHC diversity confers no advantage for phenotypic quality and reproductive performance in a wild bird. J Anim Ecol (2022) 91:1707–18. doi: 10.1111/1365-2656.13737 PMC9542035 35521665

[26] Migalska M Sebastian A Radwan J. Major histocompatibility complex class I diversity limits the repertoire of T cell receptors. Proc Natl Acad Sci U.S.A. (2019) 116:5021–6. doi: 10.1073/pnas.1807864116 PMC6421458 30796191

[27] Migalska M Sebastian A Konczal M Kotlík P Radwan J. *De novo* transcriptome assembly facilitates characterisation of fast-evolving gene families, MHC class I in the bank vole (Myodes glareolus). Heredity (Edinb) (2017) 118:348–57. doi: 10.1038/hdy.2016.105 PMC5345602 27782121

[28] Scherman K Råberg L Westerdahl H. Positive selection on MHC class II DRB and DQB genes in the bank vole (Myodes glareolus). J Mol Evol (2014) 78:293–305. doi: 10.1007/s00239-014-9618-z 24748547

[29] Bryja J Galan M Charbonnel N Cosson JF. Duplication, balancing selection and trans-species evolution explain the high levels of polymorphism of the DQA MHC class II gene in voles (Arvicolinae). Immunogenetics (2006) 58:191–202. doi: 10.1007/s00251-006-0085-6 16467985

[30] Roche PA Furuta K. The ins and outs of MHC class II-mediated antigen processing and presentation. Nat Rev Immunol 2015 154 (2015) 15:203–16. doi: 10.1038/nri3818 PMC6314495 25720354

[31] Sadowska ET Baliga-Klimczyk K Chrzaścik KM Koteja P. Laboratory model of adaptive radiation: a selection experiment in the bank vole. Physiol Biochem Zool (2008) 81:627–40. doi: 10.1086/590164 18781839

[32] Migalska M Węglarczyk K Mężyk-Kopeć R Homa J. Cross-reactivity of antibodies against T cell markers in the Bank vole (Myodes glareolus). J Immunol Methods (2023) 520:113524. doi: 10.1016/j.jim.2023.113524 37463649

[33] Channathodiyi P Houseley J. Glyoxal fixation facilitates transcriptome analysis after antigen staining and cell sorting by flow cytometry. PloS One (2021) 16. doi: 10.1371/JOURNAL.PONE.0240769 PMC7822327 33481798

[34] Crispe IN. Liver antigen-presenting cells. J Hepatol (2011) 54:357–65. doi: 10.1016/j.jhep.2010.10.005 PMC3031082 21084131

[35] Lewis SM Williams A Eisenbarth SC. Structure and function of the immune system in the spleen. Sci Immunol (2019) 4:1–12. doi: 10.1126/sciimmunol.aau6085 PMC6495537 30824527

[36] Migalska M Sebastian A Radwan J. Profiling of the TCRβ repertoire in non-model species using high-throughput sequencing. Sci Rep (2018) 8:11613. doi: 10.1038/s41598-018-30037-0 30072736 PMC6072738

[37] Biedrzycka A Sebastian A Migalska M Westerdahl H Radwan J. Testing genotyping strategies for ultra-deep sequencing of a co-amplifying gene family: MHC class I in a passerine bird. Mol Ecol Resour (2017) 17:642–55. doi: 10.1111/1755-0998.12612 27762049

[38] Sebastian A Herdegen M Migalska M Radwan J. amplisas: a web server for multilocus genotyping using next-generation amplicon sequencing data. Mol Ecol Resour (2016) 16:498–510. doi: 10.1111/1755-0998.12453 26257385

[39] Doytchinova IA Flower DR. In silico identification of supertypes for class II MHCs. J Immunol (2005) 174:7085–95. doi: 10.4049/jimmunol.174.11.7085 15905552

[40] Migalska M Przesmycka K Alsarraf M Bajer A Behnke-Borowczyk J Grzybek M. Long term patterns of association between MHC and helminth burdens in the bank vole support Red Queen dynamics. Mol Ecol (2022) 31:3400–15. doi: 10.1111/mec.16486 PMC9325469 35510766

[41] Chao A. Nonparametric estimation of the number of classes in a population. Scand J Stat (1984) 11:265–70.

[42] La Gruta NL Rothwell WT Cukalac T Swan NG Valkenburg SA Kedzierska K. Primary CTL response magnitude in mice is determined by the extent of naive T cell recruitment and subsequent clonal expansion. J Clin Invest (2010) 120:1885–94. doi: 10.1172/JCI41538 PMC2877949 20440073

[43] Logunova NN Kriukova VV Shelyakin PV Egorov ES Pereverzeva A Bozhanova NG. MHC-II alleles shape the CDR3 repertoires of conventional and regulatory naïve CD4+ T cells. Proc Natl Acad Sci U.S.A. (2020) 117:13659–69. doi: 10.1073/PNAS.2003170117/SUPPL_FILE/PNAS.2003170117.SAPP.PDF PMC7306996 32482872

[44] R Core Team. R: A Language and Environment for Statistical Computing (2020). Available at: https://www.r-project.org/.

[45] Fox J. Effect displays in R for generalised linear models. J Stat Softw (2003) 8:1–27. doi: 10.18637/JSS.V008.I15

[46] Fox J Weisberg S. Visualizing fit and lack of fit in complex regression models with predictor effect plots and partial residuals. J Stat Softw (2018) 87:1–27. doi: 10.18637/JSS.V087.I09

[47] Gabry J Simpson D Vehtari A Betancourt M Gelman A. Visualization in bayesian workflow. J R Stat Soc Ser A Stat Soc (2019) 182:389–402. doi: 10.1111/RSSA.12378

[48] Douma JC Weedon JT. Analysing continuous proportions in ecology and evolution: A practical introduction to beta and Dirichlet regression. Methods Ecol Evol (2019) 10:1412–30. doi: 10.1111/2041-210X.13234

[49] Ferrari S Cribari-Neto F. Beta regression for modelling rates and proportions. J Appl Stat (2004) 31:799–815. doi: 10.1080/0266476042000214501

[50] Brooks ME Kristensen K van Benthem KJ Magnusson A Berg CW Nielsen A. {glmmTMB} Balances speed and flexibility among packages for zero-inflated generalized linear mixed modeling. R J (2017) 9:378–400. doi: 10.32614/RJ-2017-066

[51] Hartig F. DHARMa: Residual Diagnostics for Hierarchical (Multi-Level/Mixed) Regression Models (2022). Available at: https://cran.r-project.org/package=DHARMa.

[52] Hijazi RH Jernigan RW. Modeling compositional data using dirichlet regression models. J Appl Probab Stat (2009) 4:77–91.

[53] Bürkner PC. brms: an R package for bayesian multilevel models using stan. J Stat Softw (2017) 80:1–28. doi: 10.18637/JSS.V080.I01

[54] Sharon E Sibener LV Battle A Fraser HB Garcia KC Pritchard JK. Genetic variation in MHC proteins is associated with T cell receptor expression biases. Nat Genet (2016) 48:995–1002. doi: 10.1038/ng.3625 27479906 PMC5010864

[55] Russell ML Souquette A Levine DM Schattgen SA Kaitlynn Allen E Kuan G. Combining genotypes and T cell receptor distributions to infer genetic loci determining V(D)J recombination probabilities. Elife (2022) 11. doi: 10.7554/ELIFE.73475 PMC8940181 35315770

[56] DeWitt WS Smith A Schoch G Hansen JA Matsen FA Bradley P. Human T cell receptor occurrence patterns encode immune history, genetic background, and receptor specificity. Elife (2018) 7:1–39. doi: 10.7554/eLife.38358 PMC6162092 30152754

[57] Krishna C Chowell D Gönen M Elhanati Y Chan TA. Genetic and environmental determinants of human TCR repertoire diversity. Immun Ageing (2020) 17:1–7. doi: 10.1186/s12979-020-00195-9 32944053 PMC7487954

[58] Shevyrev D Tereshchenko V Kozlov V. Immune equilibrium depends on the interaction between recognition and presentation landscapes. Front Immunol (2021) 12:706136. doi: 10.3389/fimmu.2021.706136 34394106 PMC8362327

[59] Tsai S Santamaria P. MHC class II polymorphisms, autoreactive T-cells, and autoimmunity. Front Immunol (2013) 4:321. doi: 10.3389/fimmu.2013.00321 24133494 PMC3794362

[60] Zakharova MY Belyanina TA Sokolov AV Kiselev IS Mamedov AE. The contribution of major histocompatibility complex class II genes to an association with autoimmune diseases. Acta Naturae (2019) 11:2019. doi: 10.32607/20758251-2019-11-4-4-12 PMC6977962 31993230

[61] Lenz TL Deutsch AJ Han B Hu X Okada Y Eyre S. Widespread non-additive and interaction effects within HLA loci modulate the risk of autoimmune diseases. Nat Genet (2015) 47:1085–90. doi: 10.1038/ng.3379 PMC4552599 26258845

[62] Pacholczyk R Ignatowicz H Kraj P Ignatowicz L. Origin and T cell receptor diversity of Foxp3+CD4+CD25+ T cells. Immunity (2006) 25:249–59. doi: 10.1016/J.IMMUNI.2006.05.016 16879995

[63] Georgiev P Charbonnier L-M Chatila TA. Regulatory T cells: the many faces of foxp3. J Clin Immunol (2019) 39:623–40. doi: 10.1007/s10875-019-00684-7 PMC6754763 31478130

[64] Amadori A Zamarchi R De Silvestro G Forza G Cavatton G Danieli GA. Genetic control of the CD4/CD8 T-cell ratio in humans. Nat Med (1995) 1:1279–83. doi: 10.1038/nm1295-1279 7489409

[65] Klein SL Flanagan KL. Sex differences in immune responses. Nat Publ Gr (2016) 16:626–38. doi: 10.1038/nri.2016.90 27546235

[66] Sadowska ET Stawski C Rudolf A Dheyongera G Chrząścik KM Baliga-Klimczyk K. Evolution of basal metabolic rate in bank voles from a multidirectional selection experiment. Proc Biol Sci (2015) 282:20150025. doi: 10.1098/rspb.2015.0025 25876844 PMC4426621

[67] Konczal M Koteja P Orlowska-Feuer P Radwan J Sadowska ET Babik W. Genomic response to selection for predatory behavior in a mammalian model of adaptive radiation. Mol Biol Evol (2016) 33:2429–40. doi: 10.1093/MOLBEV/MSW121 27401229

[68] Kotlík P Marková S Horníková M Escalante MA Searle JB. The bank vole (Clethrionomys glareolus) as a model system for adaptive phylogeography in the european theater. Front Ecol Evol (2022) 10:866605/XML/NLM. doi: 10.3389/FEVO.2022.866605/XML/NLM

[69] Grzybek M Sironen T Mäki S Tołkacz K Alsarraf M Strachecka A. Zoonotic virus seroprevalence among bank voles, Poland, 2002–2010. Emerg Infect Dis (2019) 25:1607–9. doi: 10.3201/eid2508.190217 PMC6649315 31310209

[70] Gomez-Chamorro A Battilotti F Cayol C Mappes T Koskela E Boulanger N. Susceptibility to infection with Borrelia afzelii and TLR2 polymorphism in a wild reservoir host. Sci Rep (2019) 9:1–12. doi: 10.1038/s41598-019-43160-3 31040326 PMC6491475

[71] Grzybek M Bajer A Bednarska M Al-sarraf M Behnke-borowczyk J Harris PD. Long-term spatiotemporal stability and dynamic changes in helminth infracommunities of bank voles (Myodes glareolus) in NE Poland. Parasitology (2015) 142:1722–43. doi: 10.1017/S0031182015001225 26442655

[72] Occhibove F McKeown NJ Risley C Ironside JE. Eco-epidemiological screening of multi-host wild rodent communities in the UK reveals pathogen strains of zoonotic interest. Int J Parasitol Parasites Wildl (2022) 17:278–87. doi: 10.1016/j.ijppaw.2022.02.010 PMC8927908 35309039

[73] Hammerbeck CD Hooper JW. T cells are not required for pathogenesis in the Syrian hamster model of hantavirus pulmonary syndrome. J Virol (2011) 85:9929–44. doi: 10.1128/JVI.05356-11 PMC3196444 21775442

[74] Guichelaar T van Erp EA Hoeboer J Smits NAM van Els CACM Pieren DKJ. Diversity of aging of the immune system classified in the cotton rat (Sigmodon hispidus) model of human infectious diseases. Dev Comp Immunol (2018) 82:39–48. doi: 10.1016/J.DCI.2017.12.026 29305168

[75] Hensel JA Khattar V Ashton R Ponnazhagan S. Characterization of immune cell subtypes in three commonly used mouse strains reveals gender and strain-specific variations. Lab Investig (2019) 99:93–106. doi: 10.1038/s41374-018-0137-1 30353130 PMC6524955

[76] Sim BC Aftahi N Reilly C Bogen B Schwartz RH Gascoigne NRJ. Thymic skewing of the CD4/CD8 ratio maps with the T-cell receptor α-chain locus. Curr Biol (1998) 8:701–4. doi: 10.1016/s0960-9822(98)70276-3 9637921

[77] Damoiseaux JG Cautain B Bernard I Mas M van Breda Vriesman PJ Druet P. A dominant role for the thymus and MHC genes in determining the peripheral CD4/CD8 T cell ratio in the rat. J Immunol (1999) 163:2983–9. doi: 10.4049/jimmunol.163.6.2983 10477560

[78] Ming Li H Hiroi T Zhang Y Shi A Chen G De S. TCRβ repertoire of CD4+ and CD8+ T cells is distinct in richness, distribution, and CDR3 amino acid composition. J Leukoc Biol (2016) 99:505–13. doi: 10.1189/JLB.6A0215-071RR PMC5338248 26394815

