# Supplementary material for: Evolutionary trade-offs constraining the MHC gene expansion: beyond simple TCR depletion model
Source: Front Immunol. 2024 Jan 8;14:1240723. doi: 10.3389/fimmu.2023.1240723 (PMC10801004; doi:10.3389/fimmu.2023.1240723)
Supplement: Supplementary file 3 [file DataSheet_1.pdf]

Supplementary Material

Evolutionary trade-offs constraining the MHC gene expansion: beyond simple TCR depletion model

Magdalena Migalska, Kazimierz Węglarczyk, Katarzyna Dudek, Joanna Homa

Correspondence: Corresponding Author: magda.migalska@uj.edu.pl

Contents

1 Supplementary Methods....2
1.1 RNA extraction from splenocyte lysates and liver fragments....2
1.2 Cell staining for a detailed cytometric analysis and gating strategy....2
1.3 MHC amplicon library preparation....3
1.4 Details on MHC genotyping....3
1.5 Details on MHC supertyping....4
1.6 TCR amplicon library preparation....4
1.7 Details on TCR repertoire analyses and size estimation....5
2 Supplementary Figures and Tables....7
2.1 Supplementary Figures....7
2.2 Supplementary Tables....18
3 References for the supplementary materials....39

1 Supplementary Methods

1.1 RNA extraction from splenocyte lysates and liver fragments

Aliquots #3 (ca. 1×10^6) of spleen cells (see *Tissue processing* section, main Materials and Methods), were lysed by gentle pipetting with 0.5 ml of RNAzol (Sigma-Aldrich) on the day of collection and preserved until further processing at -20˚C. Upon collection, liver fragments have been kept at 4˚C for tissue saturation with RNAlater (approx. 8h) and subsequently stored at -20˚C. Prior to RNA extraction, liver fragments were removed from RNAlater and homogenized in RNAzol, with either Bead Ruptor Elite, OMNI International (3-4 DEPC-treated 2.8mm beads, 3.1 m/s, 3×10s and 10s dwell time) or Bio-Gen PRO200 Homogenizer with DEPC-treated probes (2-3×10s and 10s dwell time). Subsequently, total RNA from spleens and liver cells/tissues was extracted using standard RNAzol protocol and stored at -80˚C. Both methods of homogenization yielded pure (as assessed by the Nanodrop measurements) and high quality (as assessed by gel electrophoresis) RNA.

1.2 Cell staining for a detailed cytometric analysis and gating strategy

Cells were first stained with LIVE/DEAD™ Fixable Near IR (780) Viability Kit (Invitrogen, cat# L34992, 1:1000). The staining was carried out at room temperature (RT), for 20 min (in 1ml, in conical tubes), and was followed by a wash with 10ml of PBS with 2% FBS. Subsequent staining with mAbs was performed in 96-well U bottom plates. First, ca. 0.5×10^6 cells/well were kept for 15 min in 100µl PBS with 2% FBS to block nonspecific binding, after which FITC-conjugated anti-CD4 mAb (clone GK1.5, eBioscience™, 1:100) was added to cells. Cells were stained for 30 min on ice and washed twice with PBS. Next, fixation/permeabilization was performed with Foxp3/Transcription Factor Staining Buffer Set (eBioscience™) according to the manufacturer's instructions. Briefly, cells were fixed in 200µl fixation/permeabilization working solution for 30 min at RT and then washed twice with working solution of permeabilization buffer. Washed cells were resuspended in permeabilization buffer, supplemented with 2% normal mouse and rat sera (eBioscience™) and blocked for 15 minutes at RT. After blocking, PE-conjugated anti-Foxp3 mAb (clone FJK-16s, eBioscience™, 1:20) and Alexa Fluor 647-conjugated anti CD3 mAb (clone CD3-12, Bio-Rad, 1:100) were added directly to cells and incubated for 40 min, at RT. Afterwards, cells were washes twice with permeabilization buffer and resuspended in PBS with 0.5% FBS.

Cell were analyzed on CytoFLEX cytometer. 10^4 events per sample were recorded and analyzed with CytExpert Acquisition and Analysis Software (Beckman Coulter, United States). Lymphocytes were gated according to forward and side scatter patterns, then live cells were gated based on LIVE/DEAD staining, and doublets were excluded based on FSC-A vs FSC-H pattern. Further gating of lymphocyte populations was established using FMO and non-stained control samples (1). Mean values from three technical replicates (Supplementary Dataset 2) of the following gates/quadrats (**Figure S1**) were used for the subsequent data analyses:

- Proportion of CD4+ T cells (CD3+CD4+) in lymphocytes – plot #4, upper right quadrant

- Proportion of CD8+ T cells (CD3+CD4-) in lymphocytes – plot #4, upper left quadrant

- Proportion of regulatory Foxp3+ T cells in CD4+ T cells – plot #5, upper right quadrant

Moreover, for an additional analysis that looked into proportion of CD4+Foxp3+ T cells in all lymphocytes (rather than in CD4+ T cells alone), mean values from the upper right quadrant of the **Figure S7** were taken. Raw values from the three replicates are deposited in an OSF repository, doi: https://osf.io/6wjrb/.

1.3 MHC amplicon library preparation

Each PCR reaction contained 1 μl of transcribed cDNA (see Main Methods), 1.25 μM of degenerate primer/0.75 μM of non-degenerate primer and HotStart MasterMix (QIAGEN). Moreover, each primer contained a 6-bp tag; a unique combination of forward and reverse tags allowed multiplexing of up to 72 samples (per primer pair) into one pool. PCR cycling conditions were: 95°C for 15 min, followed by 35 cycles at 95°C for 30 s, 55°C for 60 s, 72°C for 25 s, and a final extension at 72 °C for 10 min. Aliquots of PCR products were run on agarose gel to confirm amplification and then pooled in approximately equimolar quantities. Pools were separated on 1.5% agarose gel and appropriate bands were excised with Zymoclean™ Gel DNA Recovery Kit (Zymo Research). Platform-specific adaptors and additional indices marking separate pools were ligated with NEBNext Ultra™ II DNA Library Prep Kit for Illumina (New England BioLabs) with a PCR-free protocol provided by the manufacturer.

1.4 Details on MHC genotyping

MHC genotyping for each of the four markers (that is, regions amplified by a primer pair, i.e., MHC class I exon 3, MHC class II exon 2 of DQA, DQB and DRB) was based on cDNAs from two independent RNA extractions: R1 and R2. For each individual, R1 was an extract from spleen cells (i.e., aliquot #3 in the *Tissue processing* section in the main Methods) or from a liver fragment (when the amount of splenocytes did not suffice for a successful RNA extraction); R2 was an extract from a liver fragment (another liver fragment, if R1 was also liver-based). Subsequently, each of the four markers was amplified in four PCR reactions – two using R1 material as a template (i.e., R1A, R1B), and two using R2 as a template (i.e., R2A, R2B).

Upon HTS sequencing, all amplicons were processed with tools from the AmpliSAT suite (http://evobiolab.biol.amu.edu.pl/amplisat/index.php). First, sequencing reads were merged with AmpliMERGE and then de-multiplexed, clustered and filtered with AmpliSAS (2). We used the following AmpliSAS parameters for both MHC classes: MHC class I genes: for clustering – substitution errors (%) = 1; minimum dominant frequency (%) = 25; for filtering: Minimum variant depth: 3 reads (no minimum amplicon frequency threshold); discard chimeras = YES, discard frameshifts = YES. Two parameters differed between the two MHC classes: maximum number of clusters (variants) per amplicon = 100 or 15, and indel errors (%) = 0.05 or 0.001, for MHC class I or II, respectively. Mean per amplicon sequencing depth for MHC class I was 4462 reads (range: 2488-15194, SD: 1772), and for MHC class II: DQA 1869 (837-3360; 473), DQB 2200 (885-5000; 773), DRB 1643 (104-5000; 1024).

For each marker AmpiSAS outputs from the four PCRs were juxtaposed and compared (SI_Intermediate_genotyping_files.xlsx, OSF repository doi: https://osf.io/9yhfd/). Use of two independent genetic material extracts and total of four PCR replicates allowed us to accept variants with low read numbers and per amplicon frequencies (as long as they were present in two out of four amplicons), while controlling for possible cross contamination. Furthermore, for MHC class I, additional restrictions were added when calling variants for the final genotypes (SI_Features_MHCI.xlsx, OSF doi: https://osf.io/9yhfd/). This was done to exclude possible non-

classical, MHC Ib genes, which code for molecules characterized by tissue-specific expression, low polymorphism and specialized functions other than presentation of peptides to cytotoxic CD8+ T cells. Although some MHC Ib molecules supposedly take part in thymic selection and present some antigens to innate-like T cells with restricted TCR diversity (3), tissue-specific expression could cause systematic differences in gene number estimation between individuals for which only liver was used in MHC genotyping, versus those that used two tissue types (i.e., liver and spleen). To avoid such bias, we excluded (at the population level) variants that consistently were amplified only from liver or spleen, and never from both tissues. Based on this criterion we excluded 18 variants found only in spleen and 13 variants found only in liver (out of 120 variants that met our basic "two-in-four" genotyping criteria). Apart from tissue-specificity, presence of certain conserved, peptide-anchoring residues was proposed to characterize classical, MHC Ia genes (4). Our MHC class I amplicons covered five (out of nine proposed) such residues: T143, K146, W147, Y159 and Y171. Whereas these observations were based on data from a handful of species, and whether they would hold for classical MHC Ia of bank voles is unknown, we conservatively removed sequences with more than two substitutions at these positions (discarding further three variants). In these final genotypes the mean number of called MHC class I amino acid variants did not differ between the animals for which extraction from one vs two types of tissues were used for MHC genotyping (means: 11.7 vs 12.8, $t_{(22)}=-0.99$, $p=0.33$).

1.5 Details on MHC supertyping

For MHC supertyping, we used 166 MHC class I, and 59 DQA, 71 DQB, 37 DRB MHC class II sequences, which were either newly identified during the course of this study (Genbank IDs: OR424419-OR424467) or used for supertyping previously in (5). PSSs used as PBR proxies were taken from the literature, and were as follows: MHC class I - 97, 114, 116, 150, 152, 155, 156, 163 (from (6)), and for MHC class II: DQA – 11, 12, 24, 31, 32, 52, 62, 65, 66, 68, 72, 76 (5), DQB – 9, 13, 14, 26, 28, 47, 53, 56, 57, 67, 68, 70, 71, 75 (5,7,8), DRB – 11, 13, 16, 25, 26, 28, 30, 34, 56, 60, 67, 74, 86 (5,7,8). Z-values describing physicochemical proprieties of amino acids in PSSs were taken from (9), and the grouping was done via clustering with discriminant analysis of principal components (DAPC) (10), implemented in the adegenet package (11,12) (v. 2.1.7) in R (v. 4.2.0) (13). The exact supertyping procedure was described in detail in the Supplementary materials of (5,8), therefor here we only report parameters unique to this study. Number of clusters (k) was selected with help of the find.clusters() function (arguments: n.pca=100, max.n.clust=20, n.iter=5e5, n.start=500) in 25 replicate runs of clustering and upon visual inspection pf the plotted mean BIC values against k. To determine the optimal numbers of retained principal components (PCs) and Discriminant functions (DF) we used optim.a.score() (75 repeats) and xvalDapc (n.rep=100) functions. After repeated DAPC clustering, we kept: for MHC class I 15 PCs and 5 DFs, and for MHC class II: DQA - 12 PCs and 6 DFs, for DQB – 15 PCs and 6 DFs, for DRB – 8 PCs and 2 DFs. Finally, we used posterior assignments from the discriminate function analysis to assign MHC variants to the supertypes. For a few (n=5) MHC class I variants, posterior probability of supertype assignment was slightly lower than <0.5, and so we ascribed them manually to supertypes to which they belonged with the highest probability. Supertype assignments are in Supplementary Dataset 1 associated with this article (the numbering is arbitrary and does not correspond to that of (5,8)). 22 supertypes were identified for MHC class I, 12 for DQA, 11 DQB and 17 for DRB, which exactly matches number of supertypes detected in REF (5).

1.6 TCR amplicon library preparation

The protocol consist of three general stages: 1) RNA reverse transcription, 2) PCR 1 with bank-vole specific, nested primer specific to C region of TCRβ; 3) PCR 2 with indexed primers containing P5 and P7 adaptors. All primer and oligonucleotide sequences are available in (14).

In the first stage, total RNA extracted from 10^5 cells was reverse-transcribed by 5′RACE with SMARTScribe™ Reverse Transcriptase kit (TaKaRA) and a custom-made SmartNNNNa adaptor (IDT), which is a template-switch oligo containing Unique Molecular Identifier (a tag composed of 12 random nucleotides). Reaction setup consisted of two steps. First, mix 1 (20µl of RNA and 2.5µl of 10µM MyglTCRb_1 primer) was kept at 65°C for 2 min (to allow primer annealing). Next, the temperature was lowered to 42°C, and mix 2 was added to each sample (5× First-strand buffer, 1µl 20mM DTT, 2.5µl of 10µM SmartNNNNa, 3µl of 10mM dNTP mix, 1.5µl of SMARTScribe Reverse Transcriptase and 1µl of RNasin® Plus RNase Inhibitor, Promega). Total reaction volume was 40µl. cDNA synthesis was conducted at 42°C for 1h, after which samples were transferred on ice. Products of cDNA synthesis were treated with 5U USER enzyme (uracil–DNA glycosylase, NEB) for 1h at 37°C to degrade any leftover SmartNNNNa adaptor.

On the same day, whole 5'RACE reaction was purified with MinElute PCR purification kit (Qiagen) and eluted in 10µl EB. Entire cDNA was taken as a template for the second stage: PCR 1. Total reaction volume was 25µl, and apart from the cDNA it contained Q5® High-Fidelity 2× Master Mix (NEB), 0.5 µM of Smart20-mod and TCRb_3NN primers. PCR cycling conditions were: 98°C for 1 min, followed by 23 cycles of 98°C for 10s, 65°C for 20s, 72°C for 40s, and a final extension at 72°C for 4 min. PCR 1 products were purified using Agencourt AMPure XP beads (Beckman Coulter) with 1:0.6 DNA to beads ratio and DNA was eluted in 20µl of nuclease-free H_2O.

5µl of the purified product of PCR 1 was used as a template in the third stage, PCR 2. Total reaction volume was 20µl, and it contained Q5® High-Fidelity 2× Master Mix (NEB) and a combination of P5_50X and P7_70X primers (1.25µM each) containing indices that would be used for de-multiplexing of samples pooled in a sequencing run. PCR cycling conditions were as described for PCR 1, but with 12 cycles and 5min of final elongation. Products of the PCR 2 were run immediately after reaction on 1.5% agarose gel and bands of desired size (~600 bp) were purified with Zymoclean Gel DNA Recovery Kit (Zymo research). DNA was eluted in 15µl of warm EB and its concentration was measured with HS dsDNA Qubit kit (ThermoFisher Scientific).

1.7 Details on TCR repertoire analyses and size estimation

Reads processing followed the "Quantitative analysis of the bank vole TCRβ repertoire" described in (14). In brief, non-overlapping, paired-end reads were concatenated, Phred score-filtered and processed to correct sequencing errors with UMIs. Then, CDR3 sequences were extracted and summarized for each sample and Chao1 lower bound richness estimation (3) was calculated based on CDR3 clonotype abundance.

To determine appropriate sequencing depth needed for efficient UMI-based error correction (where certain level of over-sequencing is necessary) we analyzed rarefaction curves for four samples sequenced to a high coverage (>500 000 reads). We checked both the number of retrieved, error-corrected CDR3 variants (Supplementary **Figure S2**A) and Chao1 estimates of the TCR repertoire size (Supplementary **Figure S2**B) retrieved in a samples of 100 000 cells. CDR3 variant numbers started to plateau around 150 000 -200 000 reads, wears Chao1 estimates seemed to stabilize at 250 000-300 000 reads. We thus chose 300 000 reads as an optimal coverage for the subsequent analyses.

It was used as a general minimal coverage (Sup Tab. S1), and as a read sub-sampling threshold, used to control for differences in sequencing depth among amplicons.

Finally, we used Pearson's correlation to checked for relationship between T cell proportions in spleen and Chao1 TCR repertoire richness estimates of given T cell subset. Similarly, individual, between-subset TCR richness was checked for correlations. Upon confirming the latter (see Results in the main article), we checked whether this could be a technical or sampling artefact related to differences in cell viability or in overall cell numbers collected form a given individual. To that end, we checked whether summed Chao1 estimates from both CD4+ and CD8+ subset correlated with either proportion of live cells (gate P2, **Figure S1**) or recorded total cell number counted from spleens after cell collection. Neither proposed explanation was supported (correlation with live cells: r_{25}=0.09, p=0.644; correlation with cell counts: r_{17}= 0.19, p=0.442).

2 Supplementary Figures and Tables

2.1 Supplementary Figures

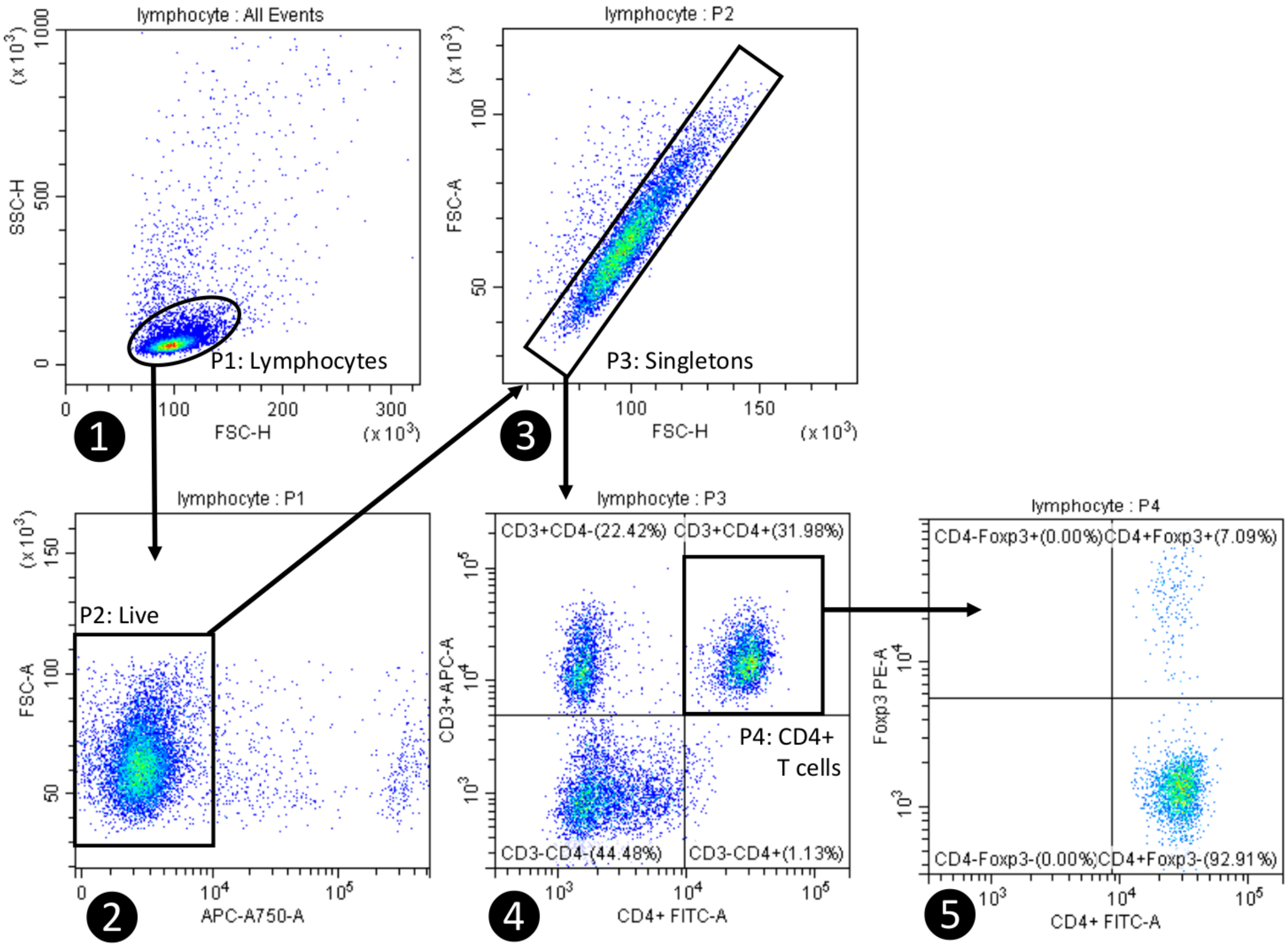

Figure S1. Gating strategy on a representative Bank vole lymphocyte staining with LIVE/DEAD™ Fixable Near IR (780) Viability Kit and following mAbs: anti-CD3 (clone CD3-12, conjugated with Alexa Fluor 647), anti-CD4 (clone GK1.5, conjugated with FITC) anti-Foxp3 (clone FJK-16s, conjugated with PE).

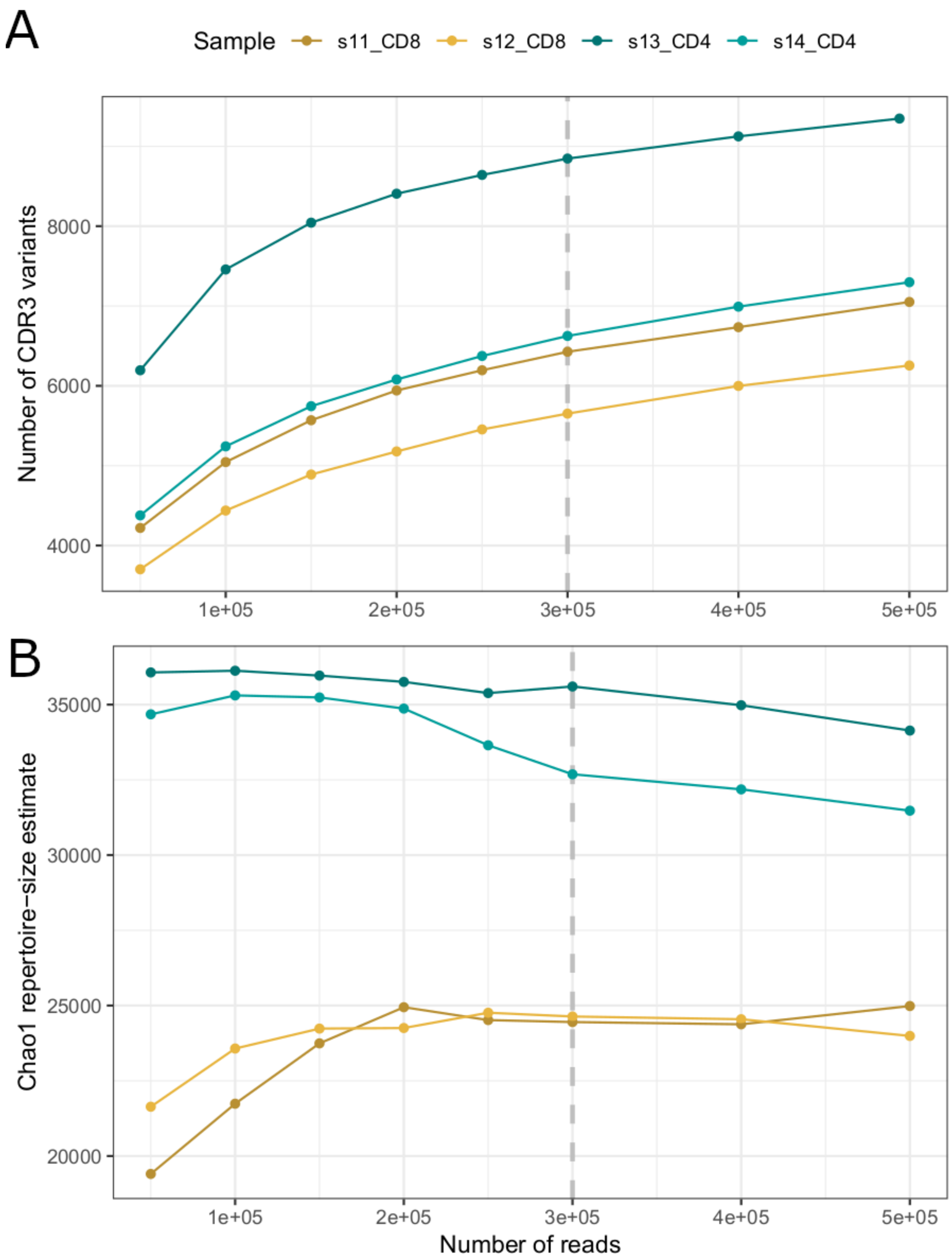

Figure S2. Rarefaction curves. Reads in four samples sequenced to a high coverage were subsampled at depths $0.5{\times}10^5$, $1{\times}10^5$, $1.5{\times}10^5$, $2{\times}10^5$, $2.5{\times}10^5$, $3{\times}10^5$, $4{\times}10^5$, $5{\times}10^5$ and either **A)** a number of retrieved, error-corrected, unique, nucleotide CDR3 variants or **B)** Chao1 estimator of TCR repertoire size was plotted. Grey, dashed, vertical line shows the value chosen for read sub-sampling in all samples.

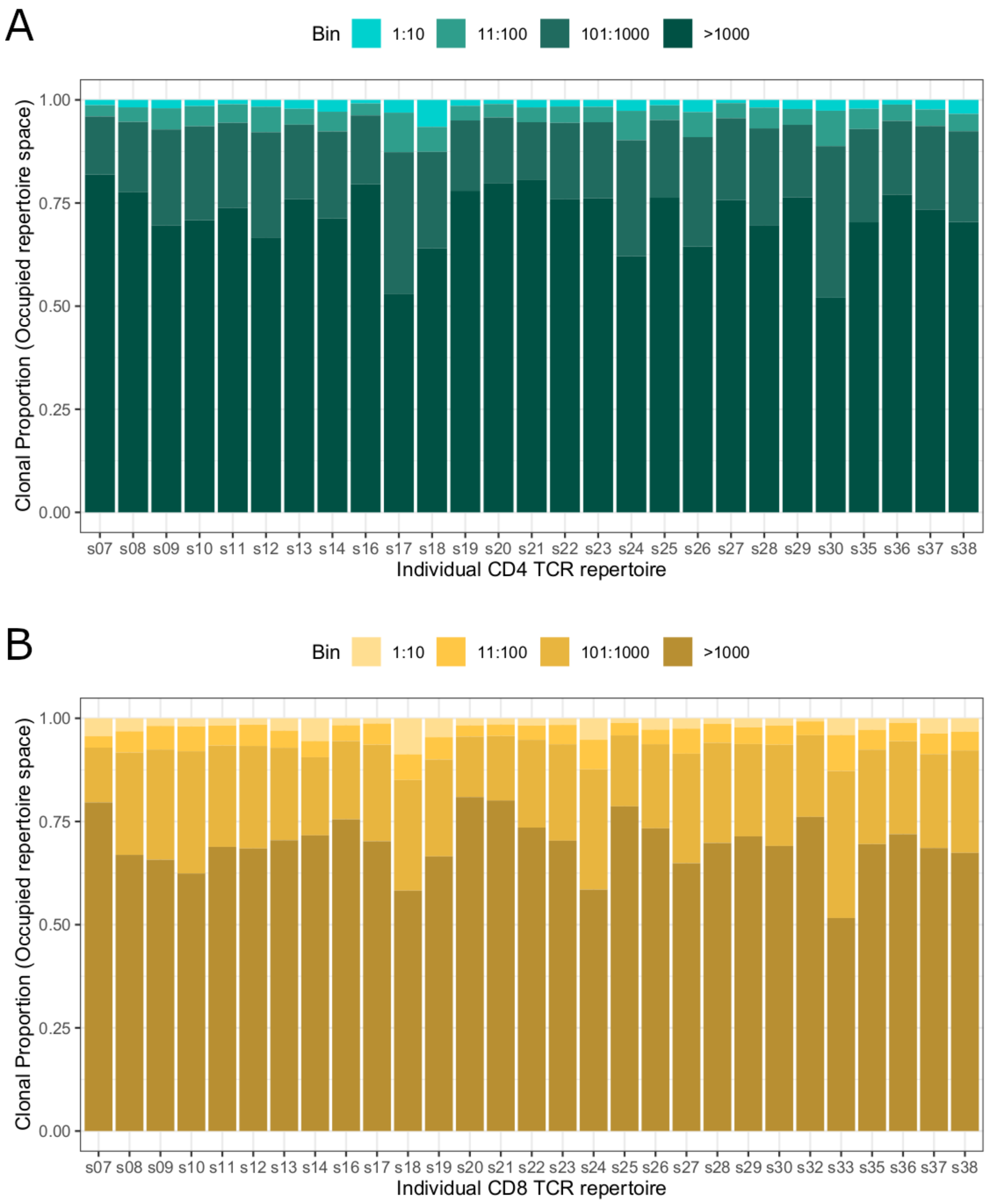

Figure S3. Clonal proportion of the top n clonotypes ranked by abundance. The brightest bin represent proportion of repertoire space occupied by the top 10 clones, the darkest bin all clones below the top 1000. Two intermediate bins represent clones ranked from 11 to 100 and from 101 to 1000.

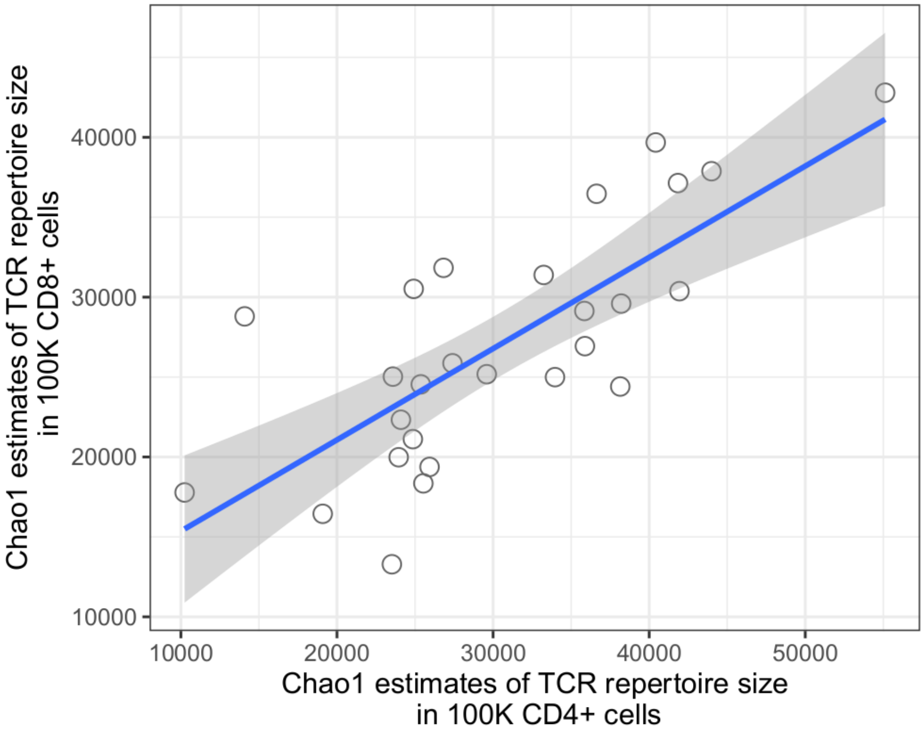

Figure S4. Correlation between individual estimates of Chao1 TCR richness in sorted CD4+ and CD8+ T cells. Blue line visualizing correlation was plotted with standard R package `ggplot2` (v 3.4.0) function stat_smooth(method="lm").

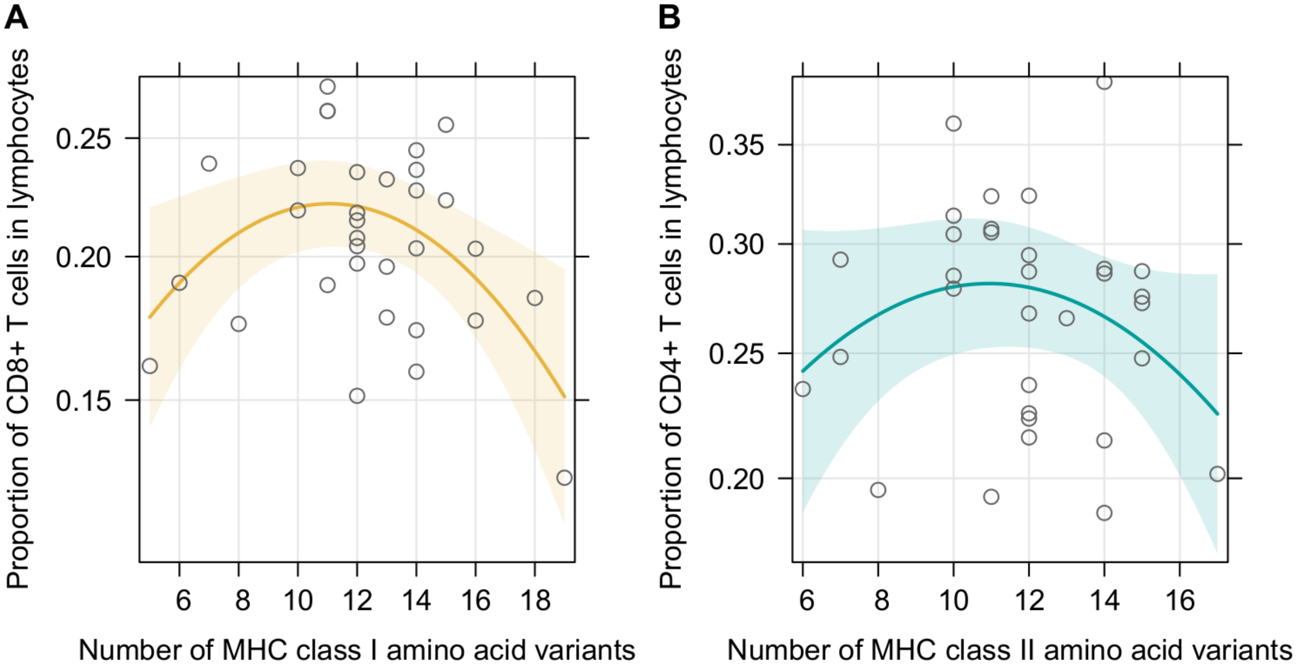

Figure S5. Predictor effect plots of A) number of MHC class I amino acid variants on the proportion of CD8+T cells among splenic lymphocytes; B) number of MHC class II amino acid variants on the proportion of CD4+T cells among splenic lymphocytes. Line shows fitted values versus the focal predictor on the horizontal axis, when the other predictor (sex) is held fixed. The shaded area is a pointwise confidence band for the fitted values, based on standard errors computed from the covariance matrix of the fitted regression coefficients. Hollow points are partial residuals.

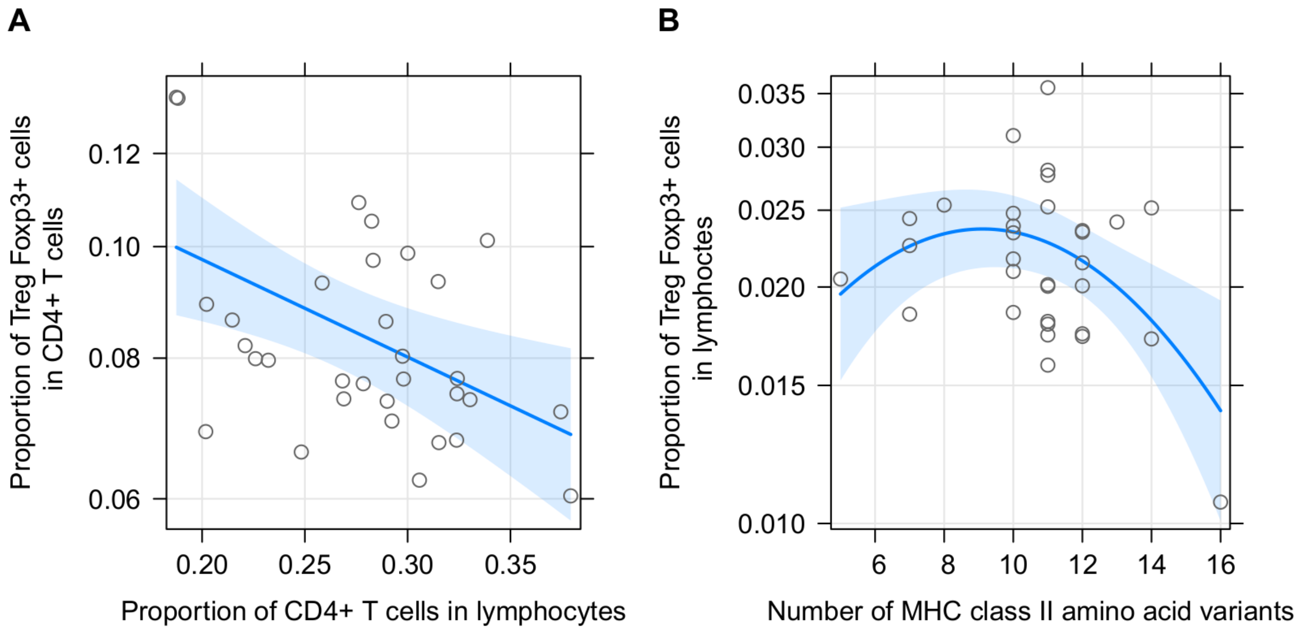

Figure S6. Predictor effect plots of A) a proportion of CD4+ T cells among splenic lymphocytes on CD4+Foxp3+ T cells within those CD4+ T cells; B) number of MHC class II supertypes on the proportion of CD4+Foxp3+ T cells among splenic lymphocytes. Blue line shows fitted values versus the focal predictor on the horizontal axis, when the other predictors (A: number of MHC class II supertypes and sex, B:sex) are held fixed. The shaded area is a pointwise confidence band for the fitted values, based on standard errors computed from the covariance matrix of the fitted regression coefficients. Hollow points are partial residuals.

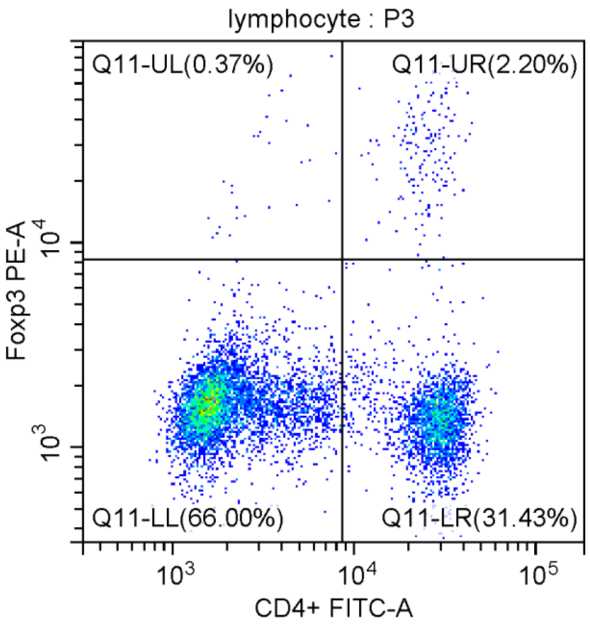

Figure S7. Representative Bank vole lymphocyte staining gated as in **Figure S1** (parent gate P3), showing anti-Foxp3 and anti-CD4 staining. Upper right quadrant shows the percentage of putative regulatory T cells among all splenic lymphocytes.

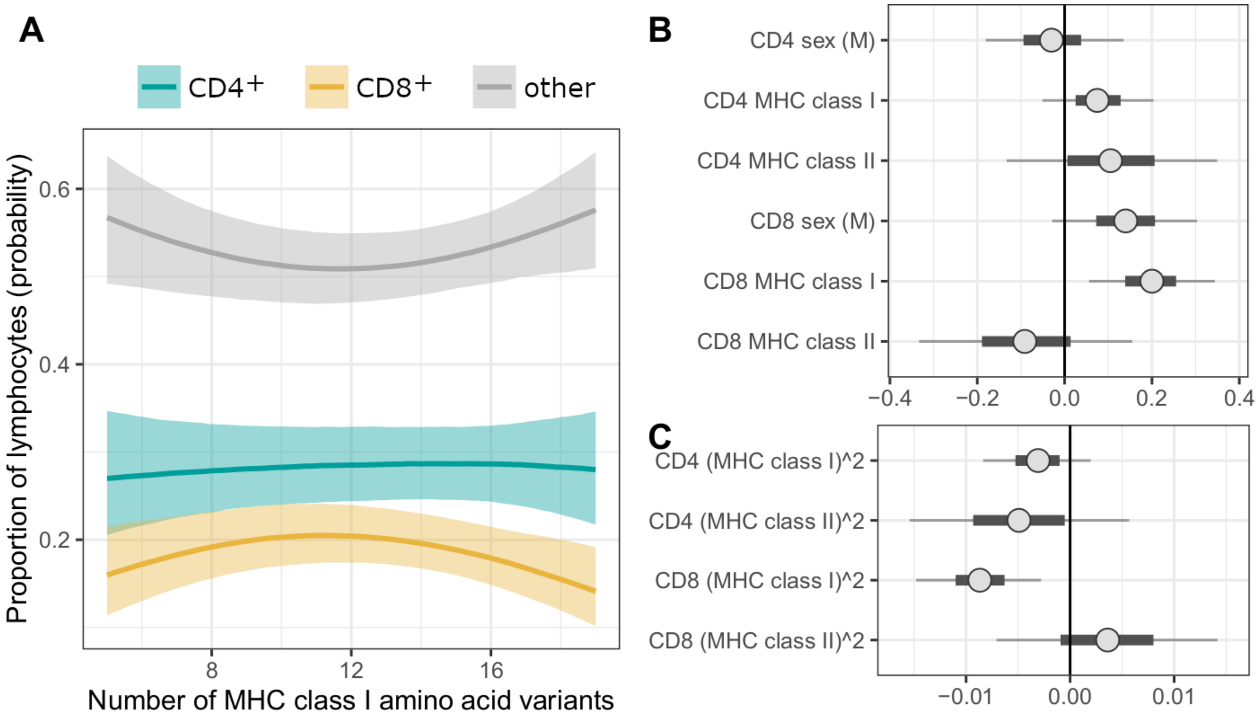

Figure S8. A) Conditional effect plot of MHC class I amino acid variant number on splenic lymphocyte proportions. The three response categories are proportion of CD4+ T cells (teal line), CD8+ T cells (yellow line) and other splenic lymphocytes (grey line). Shaded are shows 90% credible intervals (CI). B) and C) Posterior CI visualizing Markov chain Monte Carlo draws from the posterior distribution of the parameters of a Bayesian model with MHC amino acid variants and sex as predictors. "CD4" or "CD8" in the parameter prefixes corresponds to the predicted effect on given T cells subpopulation (third, reference category - "other lymphocytes" - was modelled implicitly). Figure was split into panel B (linear term) and C (quadratic term) to better visualize posterior values at different scales. The points are posterior medians, thick segments are 50% CI, thinner lines are 90% CI (package default).

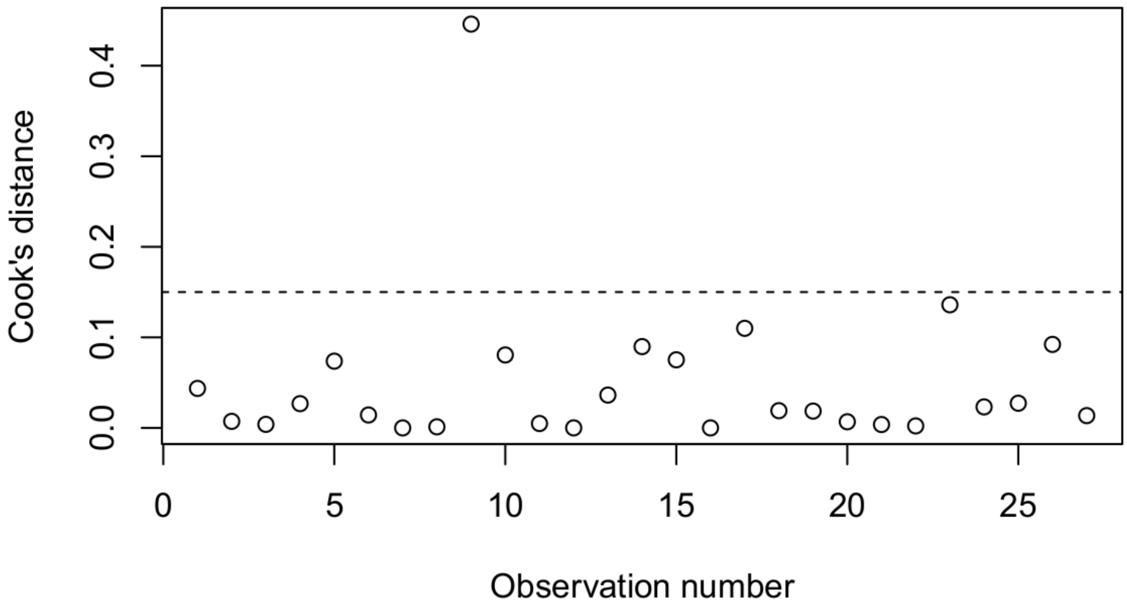

Figure S9. Cook's distances plot for a linear model predicting Chao1 TCR diversity estimates in 10^5 sorted CD4+ T cells. Explanatory variables were the number of MHC class II supertypes and sex. 9th observation (Individual s16) was identified as an influential observation in this analysis.

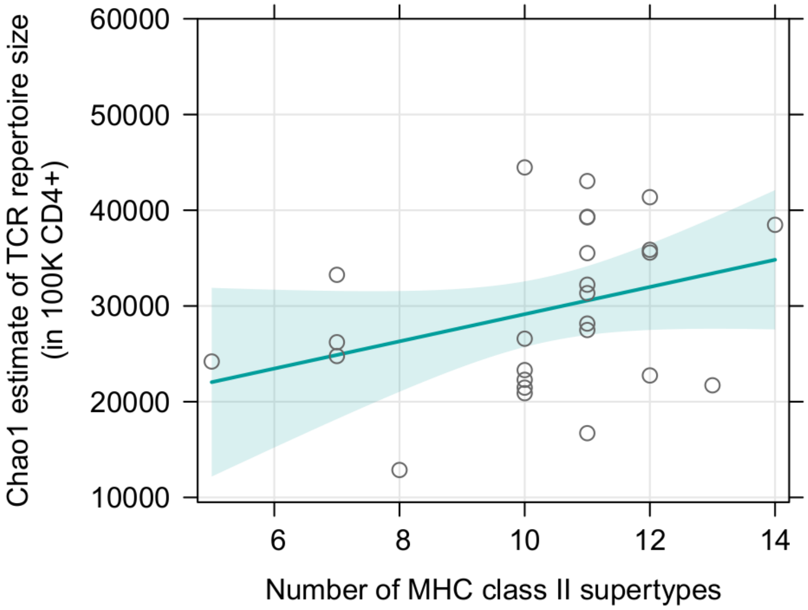

Figure S10. Predictor effect plots of number of MHC class II supertypes on the Chao1 TCR diversity estimates in 10^5 sorted CD4+ T cells – without the influential observation. Teal line shows fitted values versus the focal predictor on the horizontal axis, when other predictor (sex) was held fixed. The shaded area is a pointwise confidence band for the fitted values, based on standard errors computed from the covariance matrix of the fitted regression coefficients. Hollow points are partial residuals.

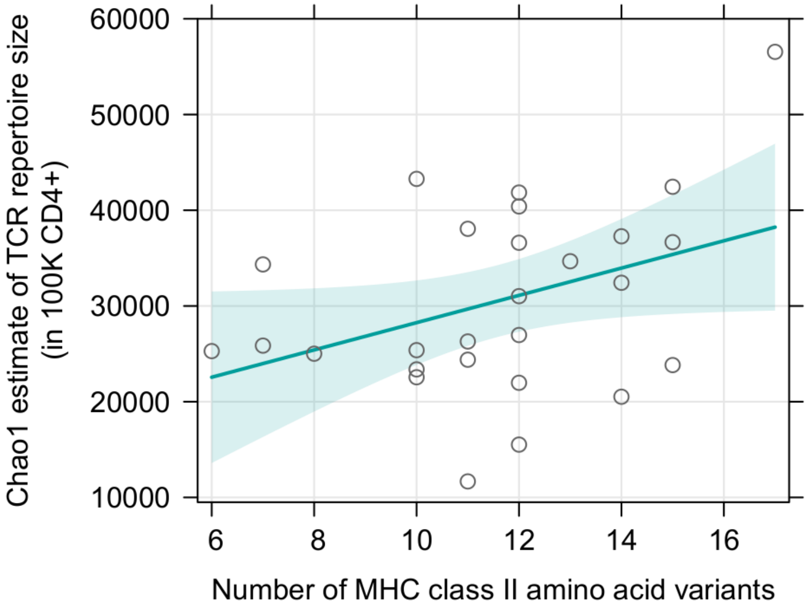

Figure S11. Predictor effect plots of number of MHC class II amino acid variants on the Chao1 TCR diversity estimates in 10^5 sorted CD4+ T cells. Teal line shows fitted values versus the focal predictor on the horizontal axis, when other predictor (sex) was held fixed. The shaded area is a pointwise confidence band for the fitted values, based on standard errors computed from the covariance matrix of the fitted regression coefficients. Hollow points are partial residuals.

2.2 Supplementary Tables

Table S1. Summary of TCR amplicon sequencing results (raw reads count: “Raw reads”, and quality-filter passing reads count: “Quality reads”), and TCR repertoire analysis, with number of observed CDR3 clonotypes (Sobs), number of clonotypes observed once (S1), twice (S2), and a lower bound Chao1 TCR richness estimation (Chao1). Ind = Individual ID.

Ind.	Cell subset	Raw reads	Quality reads	Subsampled Reads	Sobs	S1	S2	Chao1
**S07**	CD4	452287	410500	300000	13035	10466	2000	40419.29
**S07**	CD8	628207	584492	300000	13740	10818	2256	39677.31
**S08**	CD4	588182	556365	300000	10131	8449	1274	38147.33
**S08**	CD8	587788	547342	300000	6090	5136	720	24408.4
**S09**	CD4	607112	571213	300000	6815	5594	913	23952.37
**S09**	CD8	580569	548305	300000	5245	4404	658	19983.01
**S10**	CD4	550277	514202	300000	7139	5763	1014	23515.81
**S10**	CD8	424146	398384	300000	5289	3890	946	13286.94
**S11**	CD4	549056	505751	300000	8796	6790	1391	25368.29
**S11**	CD8	921023	867668	300000	6429	5377	798	24544.37
**S12**	CD4	630934	592030	300000	5715	4838	655	23582.36
**S12**	CD8	720865	669969	300000	5680	4874	614	25025.18
**S13**	CD4	719825	656832	300000	8863	7560	1059	35847.7
**S13**	CD8	611250	555528	300000	6190	5398	635	29133.63
**S14**	CD4	803323	727939	300000	6642	5835	640	33241.39
**S14**	CD8	627405	566769	300000	8258	6916	1034	31387.14
**S16**	CD4	464557	430628	300000	9443	8511	793	55115.84
**S16**	CD8	374966	340839	300000	6408	5848	470	42790.03
**S17**	CD4	352821	330732	300000	2782	2429	261	14084.76
**S17**	CD8	413412	387422	300000	5587	4913	520	28796.2
**S18**	CD4	374478	349622	300000	5291	4597	522	25532.77
**S18**	CD8	342442	323185	300000	3951	3419	406	18347.01
**S19**	CD4	356522	322460	300000	10787	8301	1832	29593.39
**S19**	CD8	544887	503084	300000	6277	5321	749	25177.56
**S20**	CD4	469723	442110	300000	10618	9061	1230	43992.68
**S20**	CD8	506569	468964	300000	14379	10902	2529	37877.14
**S21**	CD4	529734	482850	300000	13351	10716	2015	41845.46
**S21**	CD8	707419	658377	300000	10786	8900	1503	37136.63
**S22**	CD4	338165	297940	297940	9742	7479	1637	26826.74
**S22**	CD8	519568	489479	300000	7563	6476	864	31833.01
**S23**	CD4	575154	530729	300000	8817	7435	1099	33966.78
**S23**	CD8	589420	553601	300000	5467	4784	586	24994.86
**S24**	CD4	392025	369166	300000	4341	3703	465	19085.31
**S24**	CD8	381369	355151	300000	4054	3409	469	16443.43
**S25**	CD4	581126	540113	300000	8570	7382	971	36630.72
**S25**	CD8	699772	654860	300000	9556	8070	1210	36467.12
**S26**	CD4	682402	631769	300000	3825	3461	284	24913.95

**S26**	CD8	608817	572339	300000	7706	6559	943	30516.44
**S27**	CD4	484636	447113	300000	8648	6752	1406	24860.48
**S27**	CD8	577905	542708	300000	4162	3669	397	21116.11
**S28**	CD4	566015	530003	300000	5169	4581	472	27399.47
**S28**	CD8	633896	586817	300000	6091	5212	687	25861.7
**S29**	CD4	568187	530585	300000	9185	7866	1066	38206.56
**S29**	CD8	657852	614801	300000	6753	5830	744	29595
**S30**	CD4	621178	575001	300000	3035	2457	419	10238.88
**S30**	CD8	539692	502983	300000	6430	4960	1084	17777.6
**S32**	CD8	721532	668920	300000	8292	7192	878	37748.07
**S33**	CD8	376650	354866	300000	2740	2338	308	11613.77
**S35**	CD4	531008	493285	300000	7146	5802	993	24096.25
**S35**	CD8	556754	518391	300000	7106	5646	1047	22329.17
**S36**	CD4	678124	636719	300000	9567	8190	1036	41939.64
**S36**	CD8	642989	598408	300000	6527	5674	675	30374.61
**S37**	CD4	681753	634944	300000	7778	6785	819	35883.14
**S37**	CD8	647392	605097	300000	5472	4810	539	26934.06
**S38**	CD4	573582	537048	300000	7102	5908	927	25928.57
**S38**	CD8	594397	561723	300000	6145	4953	927	19377.04

Table S2. Detailed summary of MHC genotyping and supertyping results. Total – total number of variants detected in all analyzed individuals.

		Amino acid variants				Supertypes			
		total	Individual mean	range	SD	total	individual mean	range	SD
**MHC class I**		78	12.4	5-19	3.0	21	9.8	5-16	2.5
**MHC class II**	DQA	31	4.3	2-6	1.0	11	4.1	2-6	1.0
	DQB	34	4.7	2-7	1.3	10	4.2	2-7	1.1
	DRB	23	2.9	1-6	1.5	15	2.5	1-5	1.1
	sum MHC II		11.2	6-17	2.5		10.1	5-16	2.2

Table S3. GLMM coefficients for predictors of CD8+ T cell proportion in splenic lymphocytes. Fixed effects were the number of MHC class I supertypes and sex. Dispersion parameter for beta family = 228. Significance codes: *** < 0.001 < ** < 0.01 < *< 0.05 < . < 0.1

**Fixed effects**	**Estimate**	**Std. Error**	**z value**	**p value**	
(Intercept)	-1.394	0.062	-22.600	<0.001	***
sex (M)	0.110	0.066	1.685	0.092	.
MHC class I	-0.256	0.218	-1.174	0.240	
MHC class I^2	-0.403	0.203	-1.982	0.048	*

**Random effects**	**Variance**	**Std.Deviation**
Family (Intercept)	0.009	0.097
Batch (Intercept)	0.007	0.083

Table S4. GLMM coefficients for predictors of CD8+ T cell proportion in splenic lymphocytes. Fixed effects were the number of MHC class I amino acid variants and sex. Dispersion parameter for beta family = 243. LRT showed that the model with a quadratic term fitted the data better than the one without it (df=1; χ^2= 8.806; p=0.003). Significance codes: *** < 0.001 < ** < 0.01 < *< 0.05 < . < 0.1

**Fixed effects**	**Estimate**	**Std. Error**	**z value**	**p value**	
(Intercept)	-1.398	0.054	-25.802	<0.001	***
sex (M)	0.118	0.061	1.944	0.052	.
MHC class I	-0.199	0.202	-0.984	0.325	
MHC class I^2	-0.590	0.204	-2.895	0.004	**

**Random effects**	**Variance**	**Std.Deviation**
Family (Intercept)	0.009	0.096
Batch (Intercept)	0.002	0.042

Table S5. GLMM coefficients for predictors of CD4+ T cell proportion in splenic lymphocytes. Fixed effects were the number of MHC class II supertypes and sex. Dispersion parameter for beta family = 171. Significance codes: *** < 0.001 < ** < 0.01 < *< 0.05 < . < 0.1

Fixed effects	Estimate	Std. Error	z value	p value	
(Intercept)	-0.974	0.069	-14.040	<0.001	***
sex (M)	-0.063	0.067	-0.943	0.346	
MHC class II	0.153	0.236	0.646	0.518	
MHC class II^2	-0.444	0.211	-2.106	0.035	*

Random effects	Variance	Std.Deviation
Family (Intercept)	3.091×10^{-2}	1.758×10^{-1}
Batch (Intercept)	6.397×10^{-11}	7.998×10^{-6}

Table S6. GLMM coefficients for predictors of CD4+ T cell proportion in splenic lymphocytes. Fixed effects were the number of MHC class II amino acid variants and sex. Dispersion parameter for beta family = 145. LRT showed that the model with a quadratic term fitted the data marginally better than the one without it (df=1; $\chi 2$= 2.870; p=0.090). Significance codes: *** < 0.001 < ** < 0.01 < *< 0.05 < . < 0.1

**Fixed effects**	**Estimate**	**Std. Error**	**z value**	**p value**	
(Intercept)	-0.954	0.069	-13.835	<0.001	***
sex (M)	-0.086	0.074	-1.157	0.247	
MHC class II	-0.098	0.251	-0.390	0.697	
MHC class II^2	-0.388	0.221	-1.753	0.080	.

**Random effects**	**Variance**	**Std.Deviation**
Family (Intercept)	2.337×10^{-2}	1.529×10^{-1}
Batch (Intercept)	5.328×10^{-11}	7.299×10^{-6}

Table S7. GLMM coefficients for predictors of CD4+Foxp3+ T cells proportion in splenic CD4+ T cells. Fixed effects were the number of MHC class II supertypes and sex. Dispersion parameter for beta family = 413. Significance codes: *** < 0.001 < ** < 0.01 < *< 0.05 < . < 0.1

**Fixed effects**	**Estimate**	**Std. Error**	**z value**	**p value**	
(Intercept)	-2.391	0.059	-40.630	<0.001	***
sex (M)	0.037	0.067	0.550	0.585	
MHC class II	-0.392	0.221	-1.770	0.077	.
MHC class II^2	-0.134	0.204	-0.660	0.512	

**Random effects**	**Variance**	**Std.Deviation**
Family (Intercept)	1.382×10^{-2}	1.175×10^{-1}
Batch (Intercept)	6.699×10^{-11}	8.185×10^{-6}

Table S8. GLMM coefficients for predictors of CD4+Foxp3+ T cells proportion in splenic CD4+ T cells. Fixed effects were the number of MHC class II amino acid variants and sex. Dispersion parameter for beta family = 364. LRT did not support an addition of a quadratic term (df=1; $\chi 2$= 0,307; p=0.580). Significance codes: *** < 0.001 < ** < 0.01 < *< 0.05 < . < 0.1

**Fixed effects**	**Estimate**	**Std. Error**	**z value**	**p value**	
(Intercept)	-2.376	0.061	-39.140	<0.001	***
sex (M)	0.001	0.073	0.010	0.991	
MHC class II	-0.116	0.237	-0.490	0.624	
MHC class II^2	-0.119	0.216	-0.550	0.581	

**Random effects**	**Variance**	**Std.Deviation**
Family (Intercept)	1.231×10^{-2}	1.109×10^{-1}
Batch (Intercept)	6.392×10^{-11}	7.995×10^{-6}

Table S9. GLMM coefficients for predictors of CD4+Foxp3+ T cells proportion in splenic CD4+ T cells. Fixed effects were the number of MHC class II supertypes, sex and a proportion of CD4+ T cells in splenic lymphocytes. Dispersion parameter for beta family = 574. Significance codes: *** < 0.001 < ** < 0.01 < *< 0.05 < . < 0.1

**Fixed effects**	**Estimate**	**Std. Error**	**z value**	**p value**	
(Intercept)	-1.553	0.231	-6.715	<0.001	***
sex (M)	0.014	0.058	0.234	0.815	
MHC class II	-0.023	0.015	-1.552	0.121	
Proportion CD4+	-2.144	0.706	-3.035	0.002	**

**Random effects**	**Variance**	**Std.Deviation**
Family (Intercept)	1.518×10^{-2}	1.232×10^{-1}
Batch (Intercept)	3.252×10^{-11}	5.703×10^{-6}

Table S10. GLMM coefficients for predictors of CD4+Foxp3+ T cells proportion in splenic CD4+ T cells. Fixed effects were the number of MHC class II amino acid variants, sex and a proportion of CD4+ T cells in splenic lymphocytes. Dispersion parameter for beta family = 565. Significance codes: *** < 0.001 < ** < 0.01 < *< 0.05 < . < 0.1

**Fixed effects**	**Estimate**	**Std. Error**	**z value**	**p value**	
(Intercept)	-1.598	0.260	-6.155	<0.001	***
sex (M)	-0.003	0.059	-0.043	0.966	
MHC class II	-0.012	0.013	-0.889	0.374	
Proportion CD4+	-2.359	0.716	-3.296	<0.001	***

**Random effects**	**Variance**	**Std.Deviation**
Family (Intercept)	1.780×10^{-2}	1.334×10^{-1}
Batch (Intercept)	2.306×10^{-11}	4.802×10^{-6}

Table S11. GLMM coefficients for predictors of CD4+Foxp3+ T cells proportion in splenic lymphocytes. Fixed effects were the number of MHC class II supertypes and sex. Dispersion parameter for beta family = 2190. Significance codes: *** < 0.001 < ** < 0.01 < *< 0.05 < . < 0.1

**Fixed effects**	**Estimate**	**Std. Error**	**z value**	**p value**	
(Intercept)	-3.791	0.052	-72.550	<0.001	***
sex (M)	-0.023	0.055	-0.420	0.671	
MHC class II	-0.335	0.192	-1.750	0.080	.
MHC class II^2	-0.503	0.183	-2.750	0.006	**

**Random effects**	**Variance**	**Std.Deviation**
Family (Intercept)	1.426×10^{-2}	1.194×10^{-1}
Batch (Intercept)	4.703×10^{-11}	6.858×10^{-6}

Table S12. GLMM coefficients for predictors of CD4+Foxp3+ T cells proportion in splenic lymphocytes. Fixed effects were the number of MHC class II amino acid variants and sex. Dispersion parameter for beta family = 2180. LRT showed that a model with a quadratic term fitted better than the one without it (df=1; $\chi 2$= 6.535; p=0.011).
Significance codes: *** < 0.001 < ** < 0.01 < *< 0.05 < . < 0.1

**Fixed effects**	**Estimate**	**Std. Error**	**z value**	**p value**	
(Intercept)	-3.759	0.055	-68.850	<0.001	***
sex (M)	-0.086	0.058	-1.490	0.136	
MHC class II	-0.272	0.195	-1.400	0.163	
MHC class II^2	-0.476	0.181	-2.630	0.008	**

**Random effects**	**Variance**	**Std.Deviation**
Family (Intercept)	1.57×10^{-2}	1.253×10^{-1}
Batch (Intercept)	8.54×10^{-11}	9.241×10^{-6}

Table S13. Results of Bayesian analysis with Dirichlet regression, where the proportions of T cells among splenic lymphocytes was treated as a compositional proportion with three categories, i.e., CD3+CD4+ as CD4+ T cells; CD3+CD4- as CD8+ T cells; CD3-CD4- as other lymphocytes. The last category was set as a reference and model modelled implicitly. Fixed effects were sex and MHC supertypes.

**Parameter type**	**Parameter**	**T cell proportion category**	**Estimate**	**Est. Error**	**lower 95%CI**	**Upper 95%**	**Rhat**
Random effects	batch SD	CD4+	0.12	0.09	0.01	0.36	1.00
		CD8+	0.15	0.10	0.01	0.39	1.00
	faily SD	CD4+	0.23	0.11	0.05	0.48	1.00
		CD8+	0.12	0.08	0.01	0.31	1.00
Fixed Effect	Intercept	CD4+	-1.82	0.71	-3.22	-0.41	1.00
	Intercept	CD8+	-1.32	0.73	-2.80	0.08	1.00
	sex (M)	CD4+	-0.02	0.09	-0.19	0.16	1.00
	MHC class I	CD4+	0.09	0.10	-0.09	0.29	1.00
	MHC class I^2	CD4+	-0.00	0.00	-0.01	0.00	1.00
	MHC class II	CD4+	0.13	0.14	-0.15	0.39	1.00
	MHC class II^2	CD4+	-0.01	0.01	-0.02	0.01	1.00
	sex (M)	CD8+	0.12	0.09	-0.07	0.30	1.00
	MHC class I	CD8+	**0.19**	**0.10**	**-0.00**	**0.39**	**1.00**
	MHC class I^2	CD8+	**-0.01**	**0.00**	**-0.02**	**-0.00**	**1.00**
	MHC class II	CD8+	-0.09	0.14	-0.35	0.19	1.00
	MHC class II^2	CD8+	0.00	0.01	-0.01	0.02	1.00
Family-specific	phi (dispersion)		130.81	34.65	73.70	209.82	1.00

Table S14. Results of Bayesian analysis with Dirichlet regression, where the proportions of T cells among splenic lymphocytes was treated as a compositional proportion with three categories, i.e., CD3+CD4+ as CD4+ T cells; CD3+CD4- as CD8+ T cells; CD3-CD4- as other lymphocytes. The last category was set as a reference and model modelled implicitly. Fixed effects were sex and MHC amino acid variants.

**Parameter type**	**Parameter**	**T cell proportion category**	**Estimate**	**Est.Error**	**lower 95%CI**	**upper 95%**	**Rhat**
Random effects	batch SD	CD4+	0.11	0.09	0.00	0.32	1.01
		CD8+	0.12	0.09	0.01	0.32	1.00
	faily SD	CD4+	0.21	0.09	0.04	0.43	1.00
		CD8+	0.14	0.08	0.01	0.33	1.00
Fixed Effect	Intercept	CD4+	-1.61	0.81	-3.18	0.02	1.00
	Intercept	CD8+	-1.48	0.85	-3.15	0.18	1.00
	sex (M)	CD4+	-0.03	0.10	-0.21	0.17	1.00
	MHC class I	CD4+	0.08	0.08	-0.08	0.23	1.00
	MHC class I^2	CD4+	-0.00	0.00	-0.01	0.00	1.00
	MHC class II	CD4+	0.11	0.15	-0.19	0.40	1.00
	MHC class II^2	CD4+	-0.00	0.01	-0.02	0.01	1.00
	sex (M)	CD8+	0.14	0.10	-0.06	0.33	1.00
	**MHC class I**	**CD8+**	**0.20**	**0.09**	**0.03**	**0.38**	**1.00**
	**MHC class I^2**	**CD8+**	**-0.01**	**0.00**	**-0.02**	**-0.00**	**1.00**
	MHC class II	CD8+	-0.09	0.15	-0.37	0.20	1.00
	MHC class II^2	CD8+	0.00	0.01	-0.01	0.02	1.00
Family-specific	phi (dispersion)		126.04	31.55	73.61	197.16	1.00

Table S15. LM coefficients for predictors of Chao1 TCR diversity estimates in 10^5 sorted CD8+ T cells. Predictors were the number of MHC class I supertypes and sex. Residual standard error: 8265 on 26 degrees of freedom Multiple R-squared: 0.01447, Adjusted R-squared: -0.06134 F-statistic: 0.1909 on 2 and 26 DF, p-value: 0.8274. Significance codes: *** < 0.001 < ** < 0.01 < *< 0.05 < . < 0.1

**Predictors**	**Estimate**	**Std. Error**	**t value**	**p value**	
(Intercept)	27800.7	6870.4	4.046	<0.001	***
sex (M)	1652.9	3081.3	0.536	0.596	
MHC class I	-174.9	666.4	-0.263	0.795	

Table S16. LM coefficients for predictors of Chao1 TCR diversity estimates in 10^5 sorted CD8+ T cells. Predictors were the number of MHC class I amino acid variants and sex. Residual standard error: 8276 on 26 degrees of freedom Multiple R-squared: 0.01194, Adjusted R-squared: -0.06406 F-statistic: 0.1571 on 2 and 26 DF, p-value: 0.8554. Significance codes: *** < 0.001 < ** < 0.01 < *< 0.05 < . < 0.1

**Predictors**	**Estimate**	**Std. Error**	**t value**	**p value**	
(Intercept)	25778.76	6941.44	3.714	<0.001	***
sex (M)	1713.68	3076.60	0.557	0.582	
MHC class I	24.94	535.49	0.047	0.963	

Table S17. LM coefficients for predictors of Chao1 TCR diversity estimates in 10^5 sorted CD4+ T cells. Predictors were the number of MHC class II supertypes and sex. Residual standard error: 8894 on 24 degrees of freedom Multiple R-squared: 0.2491, Adjusted R-squared: 0.1865 F-statistic: 3.98 on 2 and 24 DF, p-value: 0.03215. Significance codes: *** < 0.001 < ** < 0.01 < *< 0.05 < . < 0.1

**Predictors**	**Estimate**	**Std. Error**	**t value**	**p value**	
(Intercept)	9782.6	8259.9	1.184	0.248	
sex (M)	-4846.1	3579.5	-1.354	0.188	
MHC class II	2211.0	800.6	2.762	0.011	*

Table S18. LM coefficients for predictors of Chao1 TCR diversity estimates in 10^5 sorted CD4+ T cells - dataset without the influential observation. Predictors were the number of MHC class II supertypes and sex. Residual standard error: 8344 on 23 degrees of freedom Multiple R-squared: 0.1573, Adjusted R-squared: 0.08405 F-statistic: 2.147 on 2 and 23 DF, p-value: 0.1397. Significance codes: *** < 0.001 < ** < 0.01 < *< 0.05 < . < 0.1

**Predictors**	**Estimate**	**Std. Error**	**t value**	**p value**	
(Intercept)	17562.1	8615.6	2.038	0.053	.
sex (M)	-5255.5	3364.0	-1.562	0.132	
MHC class II	1420.9	842.9	1.686	0.105	

Table S19. LM coefficients for predictors of Chao1 TCR diversity estimates in 10^5 sorted CD4+ T cells. Predictors were the number of MHC class II amino acid variants and sex. Residual standard error: 9438 on 24 degrees of freedom Multiple R-squared: 0.1544, Adjusted R-squared: 0.08398 F-statistic: 2.192 on 2 and 24 DF, p-value: 0.1336. Addition of quadratic term was not supported by LRT of nested models (df=1; F= 3.717; p=0.066). Significance codes: *** < 0.001 < ** < 0.01 < *< 0.05 < . < 0.1

**Predictors**	**Estimate**	**Std. Error**	**t value**	**p value**	
(Intercept)	15546.7	8338.5	1.864	0.075	.
sex (M)	-2973.6	3668.2	-0.811	0.426	
MHC class II	1425.2	704.9	2.022	0.055	.

Table S20. LM coefficients for predictors of Chao1 TCR diversity estimates in 10^5 sorted CD4+ T cells - dataset without the influential observation. Predictors were the number of MHC class II amino acid variants and sex. Residual standard error: 8624 on 23 degrees of freedom Multiple R-squared: 0.09989, Adjusted R-squared: 0.02162 F-statistic: 1.276 on 2 and 23 DF, p-value: 0.2981. Significance codes: *** < 0.001 < ** < 0.01 < *< 0.05 < . < 0.1

**Predictors**	**Estimate**	**Std. Error**	**t value**	**p value**	
(Intercept)	22959.8	8223.0	2.792	0.010	*
sex (M)	-4179.3	3389.3	-1.233	0.230	
MHC class II	765.1	700.5	1.092	0.286	

3 References for the supplementary materials

1. Migalska M, Węglarczyk K, Mężyk-Kopeć R, Homa J. Cross-reactivity of antibodies against T cell markers in the Bank vole (Myodes glareolus). *J Immunol Methods* (2023) 520:113524. doi: https://doi.org/10.1016/j.jim.2023.113524

2. Sebastian A, Herdegen M, Migalska M, Radwan J. amplisas: a web server for multilocus genotyping using next-generation amplicon sequencing data. *Mol Ecol Resour* (2016) 16:498–510. doi: 10.1111/1755-0998.12453

3. Chao, Anne. Nonparametric Estimation of the Number of Classes in a Population. *Scand J Stat* (1984) 11:265–270.

4. Kaufman J, Salomonsen J, Flajnik M. Evolutionary conservation of mhc class I and class II molecules - different yet the same. *Semin Immunol* (1994) 6:411–424. doi: 10.1006/smim.1994.1050

5. Migalska M, Sebastian A, Radwan J. Major histocompatibility complex class I diversity limits the repertoire of T cell receptors. *Proc Natl Acad Sci U S A* (2019) 116:5021–5026. doi: 10.1073/pnas.1807864116

6. Migalska M, Sebastian A, Konczal M, Kotlík P, Radwan J. De novo transcriptome assembly facilitates characterisation of fast-evolving gene families, MHC class I in the bank vole (Myodes glareolus). *Heredity (Edinb)* (2017) 118:348–357. doi: 10.1038/hdy.2016.105

7. Scherman K, Råberg L, Westerdahl H. Positive Selection on MHC Class II DRB and DQB Genes in the Bank Vole (Myodes glareolus). *J Mol Evol* (2014) 78:293–305. doi: 10.1007/s00239-014-9618-z

8. Migalska M, Przesmycka K, Alsarraf M, Bajer A, Behnke-Borowczyk J, Grzybek M, Behnke JM, Radwan J. Long term patterns of association between MHC and helminth burdens in the bank vole support Red Queen dynamics. *Mol Ecol* (2022) 31:3400–3415. doi: 10.1111/mec.16486

9. Sandberg M, Eriksson L, Jonsson J, Sjöström M, Wold S. New Chemical Descriptors Relevant for the Design of Biologically Active Peptides. A Multivariate Characterization of 87 Amino Acids. *J Med Chem* (1998) 41:2481–2491. doi: 10.1021/jm9700575

10. Jombart T, Devillard S, Balloux F. Discriminant analysis of principal components: a new method for the analysis of genetically structured populations. *BMC Genet* (2010) 11:94. doi: 10.1186/1471-2156-11-94

11. Jombart T. adegenet: a R package for the multivariate analysis of genetic markers. *Bioinformatics* (2008) 24:1403–1405. doi: 10.1093/bioinformatics/btn129

12. Jombart T, Ahmed I. adegenet 1.3-1: new tools for the analysis of genome-wide SNP data. *Bioinformatics* (2011) 27:3070–3071. doi: 10.1093/bioinformatics/btr521

13. R Core Team. R: A Language and Environment for Statistical Computing. (2022) https://www.r-project.org/

14. Migalska M, Sebastian A, Radwan J. Profiling of the TCRβ repertoire in non-model species using high-throughput sequencing. *Sci Rep* (2018) 8:11613. doi: 10.1038/s41598-018-30037-0
